# Expansion of GA Dinucleotide Repeats Increases the Density of CLAMP Binding Sites on the X-Chromosome to Promote *Drosophila* Dosage Compensation

**DOI:** 10.1371/journal.pgen.1006120

**Published:** 2016-07-14

**Authors:** Guray Kuzu, Emily G. Kaye, Jessica Chery, Trevor Siggers, Lin Yang, Jason R. Dobson, Sonia Boor, Jacob Bliss, Wei Liu, Gerwald Jogl, Remo Rohs, Nadia D. Singh, Martha L. Bulyk, Michael Y. Tolstorukov, Erica Larschan

**Affiliations:** 1 Department of Molecular Biology, Cellular Biology and Biochemistry, Brown University, Providence, Rhode Island, United States of America; 2 Department of Molecular Biology, Massachusetts General Hospital, Boston, Massachusetts, United States of America; 3 Department of Cell Biology, Massachusetts General Hospital Cancer Center, Boston, Massachusetts, United States of America; 4 Department of Biology, Boston University, Boston, Massachusetts, United States of America; 5 Molecular and Computational Biology Program, Department of Biological Sciences, University of Southern California, Los Angeles, California, United States of America; 6 Novartis Institutes for Biomedical Research, Cambridge, Massachusetts, United States of America; 7 Institute of Plant Protection and Microbiology, Zhejiang Academy of Agricultural Sciences, Hangzhou, China; 8 Department of Biological Sciences, North Carolina State University, Raleigh, North Carolina, United States of America; 9 Division of Genetics, Department of Medicine, Brigham and Women’s Hospital and Harvard Medical School, Boston, Massachusetts, United States of America; 10 Department of Pathology, Brigham and Women’s Hospital and Harvard Medical School, Boston, Massachusetts, United States of America; Umea University, SWEDEN

## Abstract

Dosage compensation is an essential process that equalizes transcript levels of X-linked genes between sexes by forming a domain of coordinated gene expression. Throughout the evolution of *Diptera*, many different X-chromosomes acquired the ability to be dosage compensated. Once each newly evolved X-chromosome is targeted for dosage compensation in XY males, its active genes are upregulated two-fold to equalize gene expression with XX females. In *Drosophila melanogaster*, the CLAMP zinc finger protein links the dosage compensation complex to the X-chromosome. However, the mechanism for X-chromosome identification has remained unknown. Here, we combine biochemical, genomic and evolutionary approaches to reveal that expansion of GA-dinucleotide repeats likely accumulated on the X-chromosome over evolutionary time to increase the density of CLAMP binding sites, thereby driving the evolution of dosage compensation. Overall, we present new insight into how subtle changes in genomic architecture, such as expansions of a simple sequence repeat, promote the evolution of coordinated gene expression.

## Author Summary

From stem cells to neurons, regulation of gene dosage is essential in all tissues and species in which it has been studied. Gene dosage must be balanced largely because it is critical to maintain the stoichiometry of components of multi-protein complexes that are encoded at diverse locations throughout the genome. The X-chromosome in many heterogametic species is a natural case where there is a seeming imbalance in gene dosage between sexes. How do cells correct imbalances in gene dosage? The key first step in dosage compensation across species is distinguishing the X-chromosome from the autosomes. Here we report a new mechanism by which newly evolved X-chromosomes become dosage compensated in *Diptera*: the expansion of simple GA dinucleotide repeats increases the density of the conserved CLAMP zinc finger protein that recruits the dosage compensation complex to the X-chromosome. Expansion of dinucleotide repeats can easily occur via slippage of DNA polymerase providing a simple mechanism for nucleating a domain of coordinated gene expression.

## Introduction

Changes in primary DNA sequence that occur over evolutionary time alter transcription factor occupancy on DNA [1,2]. Differential occupancy of transcription factors throughout the genome controls the essential gene regulatory programs that define growth and development [3]. Sex chromosome dosage compensation is a key model system with which to study this essential process because a large number of genes on a single chromosome are co-regulated to form a domain of coordinated gene expression [4,5]. However, little is understood about the evolutionary mechanisms that drive the differentiation of the sex chromosomes to ensure the specificity of this new domain.

Recent work demonstrated that the same mechanism of dosage compensation evolved independently across Dipterans, although the diverged sex chromosomes are not all derived from the same ancient chromosome [6]. This conserved mechanism increases the transcript levels of all active genes along the length of the male X-chromosome two-fold to equalize gene expression between males (XY) and females (XX) [6,7]. Because many different chromosomes evolved the same mechanism independently, we hypothesized that the ability of any *cis*-acting DNA sequences involved in this process to be easily generated is critical. For the past thirty years, it has been known that dinucleotide repeats are enriched on the *Drosophila* X-chromosome [8]. It was hypothesized that these repeats promote targeting of the dosage compensation machinery to the X-chromosome [8], yet the mechanism remained unknown.

In *Drosophila melanogaster*, the best studied *Dipteran* species, dosage compensation is mediated by the MSL (Male-Specific Lethal) complex that deposits H4K16ac, which is likely to increase transcript levels by opening chromatin to allow more rapid progression of RNA Polymerase II through gene bodies[7,9–11]. The MSL complex includes five protein components and two non-coding RNAs [12] and is recruited to X-linked Chromatin Entry Sites (CES) by 21-bp GA-rich DNA sequence elements called MREs (MSL Recognition Elements) [13]. MREs are 1.8 fold enriched on the X-chromosome compared with autosomes in both *D*. *melanogaster* and the distantly related *D*. *miranda*. Moreover, MREs have been acquired on the newly evolved *D*. *miranda* neo X-chromosome that is only 1 million years old compared with the ancient X-chromosomes that are 30 million years old [14]. The enrichment of MREs on the X-chromosome over evolutionary time likely occurred via a combination of transposon insertions, gene conversion, and errors in DNA replication [15–17].

Interestingly, the 21-bp MREs are much longer than most transcription factor binding sites that are on average 6 to 8-bp in length (http://the_brain.bwh.harvard.edu/uniprobe). Not only do MREs have an 8-bp highly conserved core sequence, but they also contain more degenerate flanking sequence outside of the core motif that is required for MSL complex recruitment [13]. Canonical MSL complex components do not directly interact with the MRE in a sequence specific manner, other than a low affinity interaction between the MSL2 protein and a single cytosine within the MRE[18,19]. Therefore, the mechanism by which the complete 21-bp MRE motif promotes MSL recruitment to the X-chromosome remained poorly understood.

We recently demonstrated that the CLAMP C2H2 zinc finger protein directly recognizes MRE sequences and is required for MSL complex recruitment, thereby providing the first link between MSL complex and the X-chromosome [20]. When CLAMP and MSL complex are colocalized, the occupancy of both factors is increased likely due to: 1) the deposition of the H4K16ac mark by MSL complex that opens chromatin to allow CLAMP to identify its binding sites more efficiently [9,20] and 2) physical association between CLAMP and MSL complex [21]. Moreover, the CLAMP protein occupies MREs on both the X-chromosome and autosomes and is enriched on the X-chromosome even in the absence of MSL complex [20]. Therefore, it is key to understand how the binding sites for CLAMP became enriched on the X-chromosome to promote specific X-identification for dosage compensation.

Here, we integrate biochemical, *in vivo*, and evolutionary approaches to provide new insight into how the X-chromosome likely evolved as a domain of coordinated gene regulation. First, CLAMP occupancy increases as the number of GA-repeats increases, a feature that provides a possible mechanism for easily generating new high affinity CLAMP binding sites. Second, the overall density of CLAMP occupancy is highest within the MSL complex CES, and the location of CLAMP binding sites relative to genes differs on the X-chromosome compared to autosomes, increasing X-specificity. Third, CLAMP and its DNA binding sequence are enriched on the X-chromosome across several distantly related species. Therefore, we provide support for a mechanism by which expansion of simple sequence repeats over evolutionary time increases the density of CLAMP within CES to drive the X-specificity of dosage compensation.

## Results

### CLAMP directly binds to a long GA-rich motif that is enriched at *in vivo* targets

**Fig 1:**
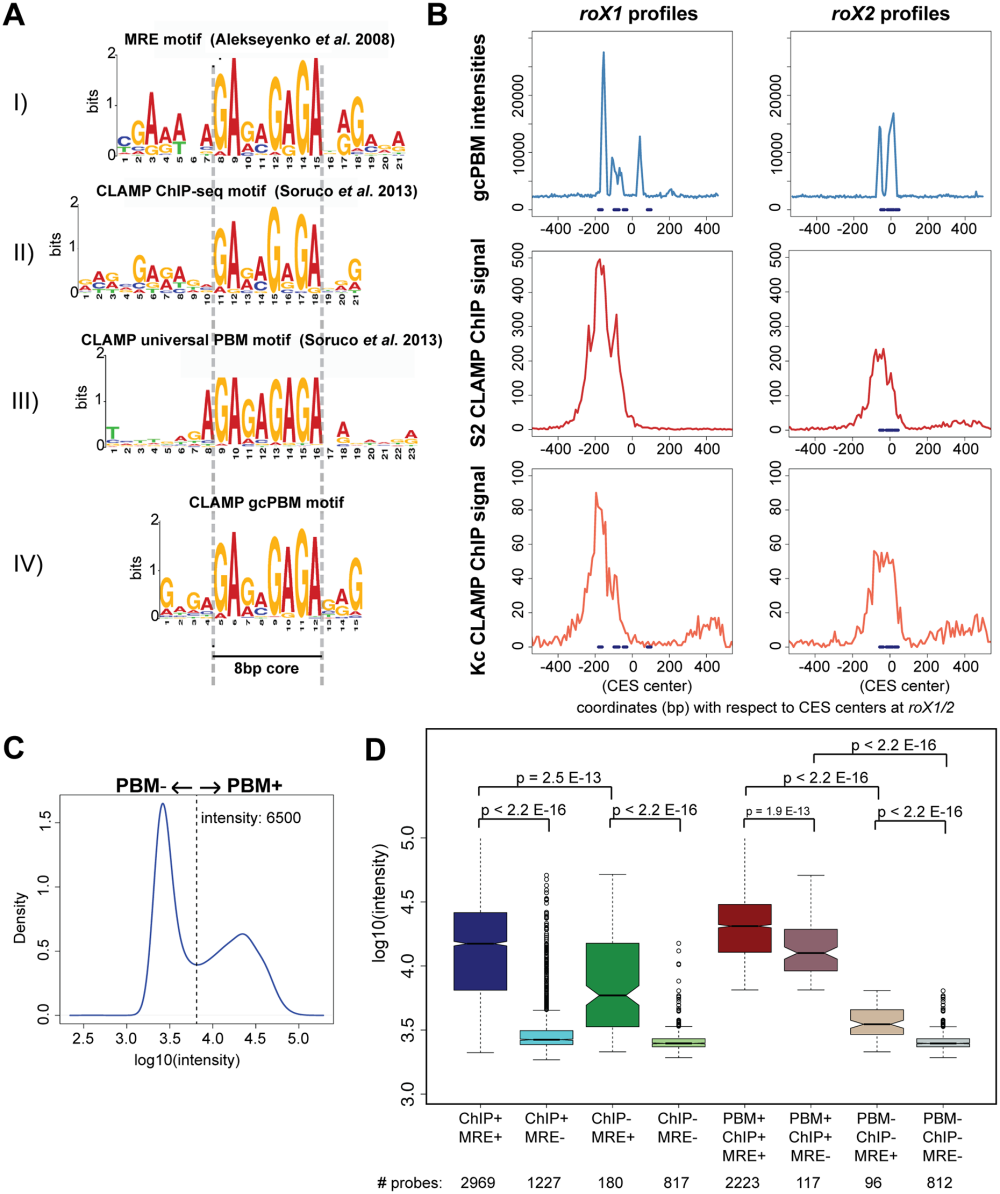
CLAMP directly binds to a long GA-rich motif that is enriched at *in vivo* targets. **A)** The following motifs are shown: I) MRE motif [13]. II) CLAMP *in vivo* motif derived from ChIP-seq data from S2 cells [20]; III) CLAMP *in vitro* motif derived from the uPBM [20] and IV) CLAMP *in vitro* motif that we derived from the custom gcPBM. The most conserved part of the motifs, their 8-bp core, is highlighted between two dashed lines. **B)** gcPBM intensities (intensity from genomic context PBM) (top) and CLAMP ChIP signal (input normalized RPKM: Reads per Kb per Million) for S2 (middle) and Kc (bottom) cells are plotted for 800 bp windows centered at each of two MSL complex CES (*roX1*, left, and *roX2*, *right*). MRE sequences are shown as blue dashes on each profile. **C)** A histogram of intensities from the gcPBM experiment. The intensity of 6500, between the two peaks of the bimodal distribution, is indicated with a dashed line. Probes with intensities higher than 6500 are designated PBM+ and those that are lower than 6500 are considered PBM-. **D)** Box plots are used to compare the intensities for probes sorted by whether they contain sequences that are bound or unbound from *in vivo* ChIP-seq data (ChIP +/-) and whether or not they conform to the previously characterized MRE motif (MRE+/-). In the right half of the panel, probes were resorted to add the category of bound or unbound on the PBM (PBM+/-). p-values for comparisons between categories are displayed above the relevant boxes, and the number of probes for each category are listed for each group. For each box plot used throughout this study, the collared box indicates that 95% percent confidence interval. If the notches at the center of the box are not overlapping, it indicates that two samples are statistically different from each other.

Previously, we determined that the sequences flanking the 8-bp core of the 21-bp MRE are necessary for MSL recruitment [13] (Fig 1A: I). It was surprising that such an unusually long motif was required for MSL complex recruitment. Similarly, the CLAMP motif determined by our previous ChIP-seq analysis features an 8-bp core that is part of a longer GA-rich motif that is similar to the MRE [20] (Fig 1A: II). Therefore, we hypothesized that bases flanking the 8-bp core contribute to MSL recruitment because they are required for CLAMP binding, which then tethers the MSL complex to the X- chromosome.

To determine the requirements for CLAMP binding directly to DNA, we compared *in vivo* and *in vitro* binding properties. We previously used a universal Protein Binding Microarray (uPBM) that contains all possible combinations of 10-mer sequences and additional flanking bases within a 36-bp variable probe sequence [20]. The uPBMs identified only a short 8-bp motif (Fig 1A: III) because they were not powered to determine the role of flanking DNA sequences due to insufficient coverage of longer binding sequences [20,22]. To better recapitulate *in vivo* binding, we therefore designed a custom genomic-context PBM (gcPBM) experiment [23] using sequences derived from CLAMP ChIP-seq binding sites (ChIP+) and control sequences not bound by CLAMP *in vivo* (ChIP-) (S1 Table). We expressed a GST-CLAMP fusion protein containing the predicted 6 tandem C-terminal zinc fingers of CLAMP for these analyses (a.a. 350–561) using *in vitro* transcription and translation as in our previous studies[20].

To validate our gcPBM approach, we included control sequences on the array to compare with *in vivo* ChIP-seq binding profiles. First, we plotted the *in vitro* binding of CLAMP over two tiled regions which surround strong binding sites for MSL complex (*roX1* and *roX2*) (Fig 1B: top). We observed specific interaction with most sequences containing previously identified MREs and the gcPBM binding profiles were similar to those from previously generated *in vivo* ChIP-seq data (Fig 1B: middle and bottom). In order to extend our analysis beyond two specific locations in the genome, we next defined the CLAMP bound sequences on the gcPBM (PBM+) as those with intensities greater than 6500 based on finding the local minimum in a histogram of all of the intensities (Fig 1C). Motifs were generated by MEME analysis from the bound sequences (Fig 1A: IV). Using the gcPBM, we captured additional flanking sequences outside the 8-bp core region compared with the uPBM (Fig 1A: IV vs. 1A: III). Therefore, we demonstrated that the binding of CLAMP derived from custom gcPBMs largely matches previously observed *in vivo* data, validating our approach.

Next, we compared *in vitro* CLAMP binding to sequences that are bound (ChIP+) or not bound (ChIP-) *in vivo*. *In vitro* binding of CLAMP is significantly increased for DNA sequences that are bound *in vivo* (ChIP+ MRE+, dark blue and dark red boxes) compared to those that are not bound *in vivo* (ChIP- MRE+, dark green and tan boxes) even if both DNA sequences match the MRE that we previously derived from high affinity MSL complex binding sites[13] (Fig 1D, full set of p-values are reported in S2 Table). The presence of a sequence that matches the MRE motif further increases CLAMP binding intensity compared to those sequences without an MRE motif even if both are bound *in vivo* (Fig 1D: ChIP + MRE+ (dark blue box) vs. ChIP+ MRE- (light blue box)). MREs promote CLAMP binding for both PBM+ and PBM- sites (Fig 1D: right). In conclusion, these data suggest that sequences that CLAMP binds more strongly *in vitro* are more likely to be bound *in vivo*.

### CLAMP binding requires DNA elements flanking the 8-bp core part of the motif

**Fig 2:**
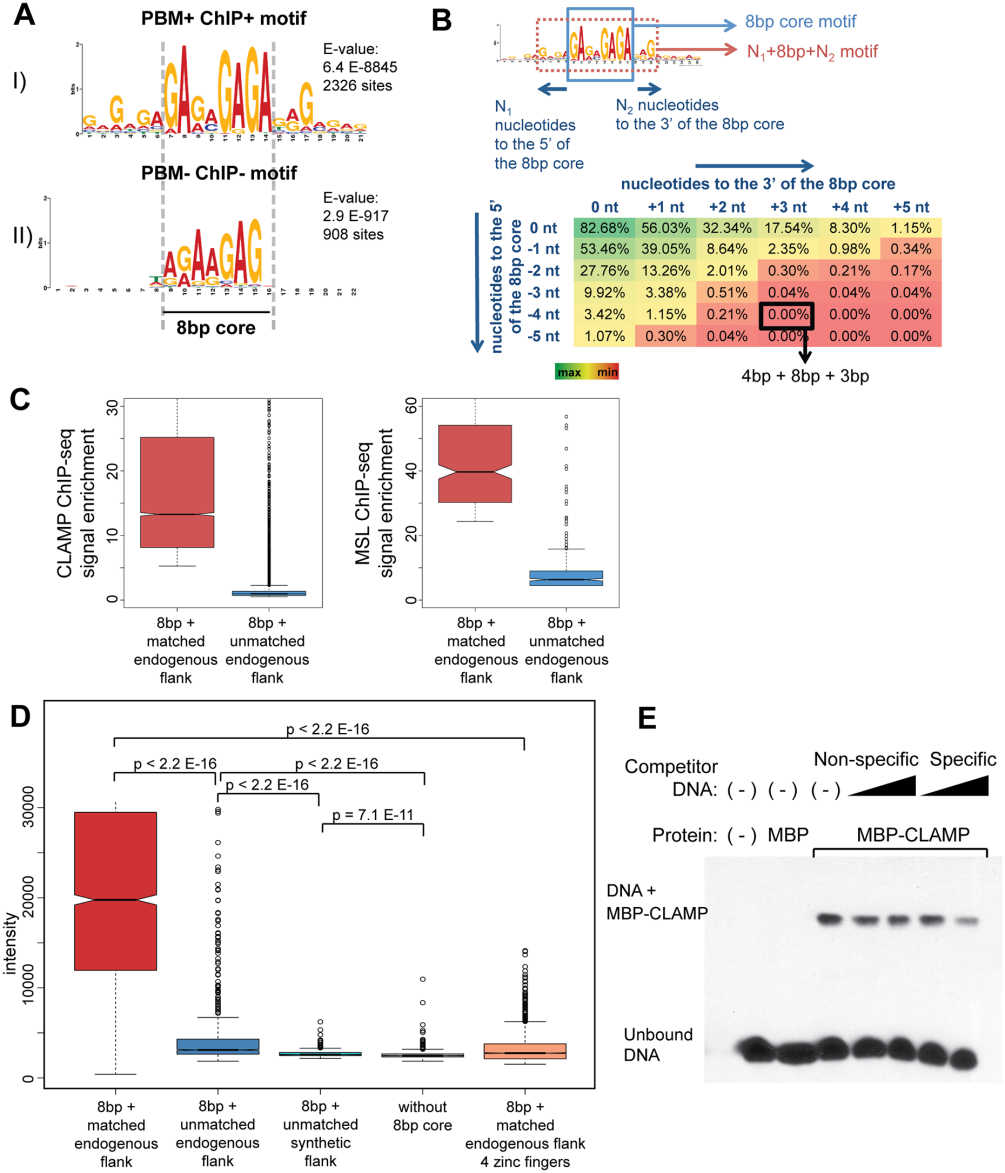
A long 15-bp motif increases CLAMP binding to DNA. **A)** Motifs obtained from the custom gcPBMs are shown: I) The PBM+ChIP+ motif represents the sequences that CLAMP binds both *in vivo* and *in vitro*. II) The PBM-ChIP- motif represents the sequences that are on the array but not bound by CLAMP *in vivo* or *in vitro*. The most conserved 8-bp core element is indicated by vertical dashed lines. **B)** A representation of the methodology to define the minimal CLAMP-bound motif by scanning both 5’ and 3’ of the core motif. The table shows the percentage of PBM+ChIP+ (CLAMP binding both *in vitro* and *in vivo*) sequences that overlap with PBM-ChIP- sequences (CLAMP binding neither *in vitro* nor *in vivo*) at the specified motif size. The y-axis shows the nucleotides 5’ of the 8-bp core motif and the x-axis shows the nucleotides 3’. The scale ranges from green for the maximal values to red for the minimal values. Values show the percentage of PBM+ChIP+ sequences shared with PBM-ChIP- sequences for the length window selected. A value of zero overlapping sequences represents complete separation between PBM+ChIP+ and PBM-ChIP- sequences and is obtained at 4-bp 5’ and 3-bp 3’ of the core motif. **C)** CLAMP and MSL ChIP-seq enrichments are shown for sequences containing the 8-bp core with and without additional flanking sequence matching the motif. Motif hits were found using the FIMO tool (p < E-4). All 8-bp core hits were found first and the ones overlapping with the full 15-bp motif were separated as ‘8bp + matched endogenous flank’ and the rest were grouped as ‘8bp + unmatched endogenous flank’. Since the ‘8bp + unmatched endogenous flank’ group has ~10,000 sites, the top 10,000 enrichments are shown in the CLAMP enrichment plot. Since there are ~300 CES, the top 300 enrichments are shown in the MSL enrichment plot. **D)** Binding intensities are shown for the following classes of probes: 1) probes with the optimal motif (8-bp + matched endogenous flank, red); 2) probes that have matching 8-bp core regions but the endogenous flanks do not match the motif (8-bp + unmatched endogenous flanks, blue); 3) probes that have 8-bp cores with synthetic constant flanking sequences (8-bp + unmatched synthetic flank, cyan); 4) probes that do not have the 8-bp core motif (without 8-bp, brown); 5) Intensities for C-terminal 4 zinc finger GST fusion proteins are shown for probes containing the 15-bp CLAMP motif (15–bp, 4ZF, orange). **E)** CLAMP binds to DNA containing a high affinity, 8 bp + matched flank motif in an electrophoretic mobility shift assay. Biotin-labeled DNA alone (lane 1) and DNA with MBP (lane 2) do not shift, while MBP-CLAMP forms a complex with DNA to shift the signal. This was competed away with specific (high affinity) competitor but not a non-specific competitor that contains the 8-bp core but lacks endogenous flanking sequences (8-bp + unmatched synthetic flank).

To determine which parts of the CLAMP motif are most critical for CLAMP binding, we used several different approaches. First, we used MEME to derive motifs from two different classes of sites: 1) PBM+ ChIP+ (**bound** *in vivo* and *in vitro*, Fig 2A: I); 2) PBM- ChIP- (**unbound** *in vivo* and *in vitro*, Fig 2A: II) (See S1A Fig for motifs from additional classes of sites). As expected, we noted overall similarities in the core of the motif because many of our probes contain the short core motif identified from our original uPBMs to allow detailed analysis of the flanking regions (see Methods). We found that DNA elements that are not bound have very little sequence flanking the core 8-bp of the motif compared with bound sites.

Next, we quantitatively determined the optimal size of the CLAMP binding site. We found the minimal number of consensus nucleotides outside of the 8-bp core that were required to separate all bound sequences (PBM+ ChIP+) from all unbound sequences (PBM-ChIP-) (Fig 2B, percent overlap of 0%). Using this approach, we identified the minimal key flanking regions as 4-bp 5’ and 3-bp 3’ of the 8-bp core sequence for a total of a 15-bp motif. After defining the 15-bp optimal CLAMP binding motif, we tested whether the ability of a sequence to match this motif correlates with increased *in vivo* CLAMP and MSL complex ChIP-seq occupancy [13,20]. We found that the presence of the 15-bp motif (8-bp + matched endogenous flank) greatly increased both CLAMP and MSL complex occupancy compared with the 8-bp core motif alone lacking any flanking sequence that matches the motif (8-bp + unmatched endogenous flank) (Fig 2C). Furthermore, we determined that the better a sequence matches the motif (decreased p-value), the higher the *in vivo* occupancy at this motif (S1B Fig, S3 Table).

Next, we measured the role of flanking sequences in a different way by dividing the bound sequences into five different quantiles based on their *in vitro* binding intensities (S2A Fig). To quantify these differences, we measured the Euclidean distances between the top quantile of bound motif instances (q1) and the bottom quantile of bound motif instances (q5) for the 8-bp core (blue) and the flanking sequences (red) (S2B Fig, S4 Table). Differences between the strongly and weakly bound motif instances were greater in the flanking region (red) compared to the core (blue), indicating that appropriate flanking bases correlate with enhanced CLAMP interaction with DNA.

To directly test the requirement for flanking sequences to enhance CLAMP binding, we compared the binding of CLAMP to probes containing the optimal 15-bp motif (8-bp + matched endogenous flank) with binding to two additional classes of probes: 1) Probes with the same 8-bp cores as those in the 15-bp motif probes but different flanks derived from endogenous sequences (8-bp core + unmatched endogenous flank); 2) Probes with variable 8-bp cores that all match the original uPBM motif and a single non-endogenous artificial constant flanking sequence (8-bp + unmatched synthetic flank). The probes containing the original 15-bp motifs (8-bp + matched endogenous flank) were bound much more strongly than either class of probes that only contained sequences matched to the 8-bp motif core (Fig 2D, p-values for all comparisons are reported as S5 Table).

Although the gcPBM was performed with a GST-CLAMP fusion as has been done previously[24], GST-tags can form homodimers potentially influencing binding profiles[25]. In order to determine whether the CLAMP motif requirements are specific to GST-tagged CLAMP, we next tested CLAMP binding using a maltose binding protein (MBP) epitope tag by electrophoretic mobility shift assay (EMSA). We observed that a gcPBM high affinity 15-bp motif-containing sequence was better able to compete for CLAMP compared with a nonspecific gcPBM sequence with constant synthetic flank sequence (Fig 2E). Therefore, a long 15-bp motif improves the interaction between CLAMP and DNA independent of the GST-CLAMP fusion.

Next, we hypothesized that the presence of a large DNA binding domain containing six tandem zinc fingers allows CLAMP to recognize a long 15-bp motif. Each DNA-binding zinc finger typically recognizes 3-bp of DNA[26] suggesting that five of the six CLAMP zinc fingers would be necessary to recognize a 15-bp motif. To further map the CLAMP DNA interaction domain, we produced constructs with one through five zinc fingers (from both the N-terminal and C-terminal directions). However, the only additional construct that produced soluble protein was the C-terminal four finger construct (a.a. 412–561). Both the six finger and four finger constructs are able to bind to DNA (Fig 2D). However, the six zinc finger protein bound to probes with the 15-bp motif (red box) with much stronger affinity than the four finger construct (orange box) (Fig 2D). This indicates that although the four fingers tested in this construct are sufficient for DNA binding, one of the two deleted fingers is required to significantly increase the affinity of CLAMP binding to probes. It is possible that a specific number of fingers (greater than four) is required, or it may be that one or both of the deleted fingers have the most specificity for the flanking sequence. In summary, we used gcPBMs to rigorously show that a long MRE, such as that previously observed to improve MSL binding [13] is required for CLAMP binding to DNA.

### CLAMP binds more efficiently to long GA-dinucleotide repeats *in vivo* and *in vitro*

Many transcription factors use the biophysical properties of DNA shape to recognize their binding sites in addition to just primary DNA sequence[27,28]. Therefore, we tested structural features of the MRE and CLAMP motifs such as minor groove width, helix twist, roll, and propeller twist[29] for their contribution to CLAMP binding. We found that specific DNA shape features did not influence CLAMP binding at its strong binding sites but there was a modest role for shape at weak binding sites (S3 Fig). Therefore, shape plays a more significant role in CLAMP binding at its weak binding sites consistent with their lack of a strong consensus motif and similar to the shape requirements observed for other transcription factors [30].

**Fig 3:**
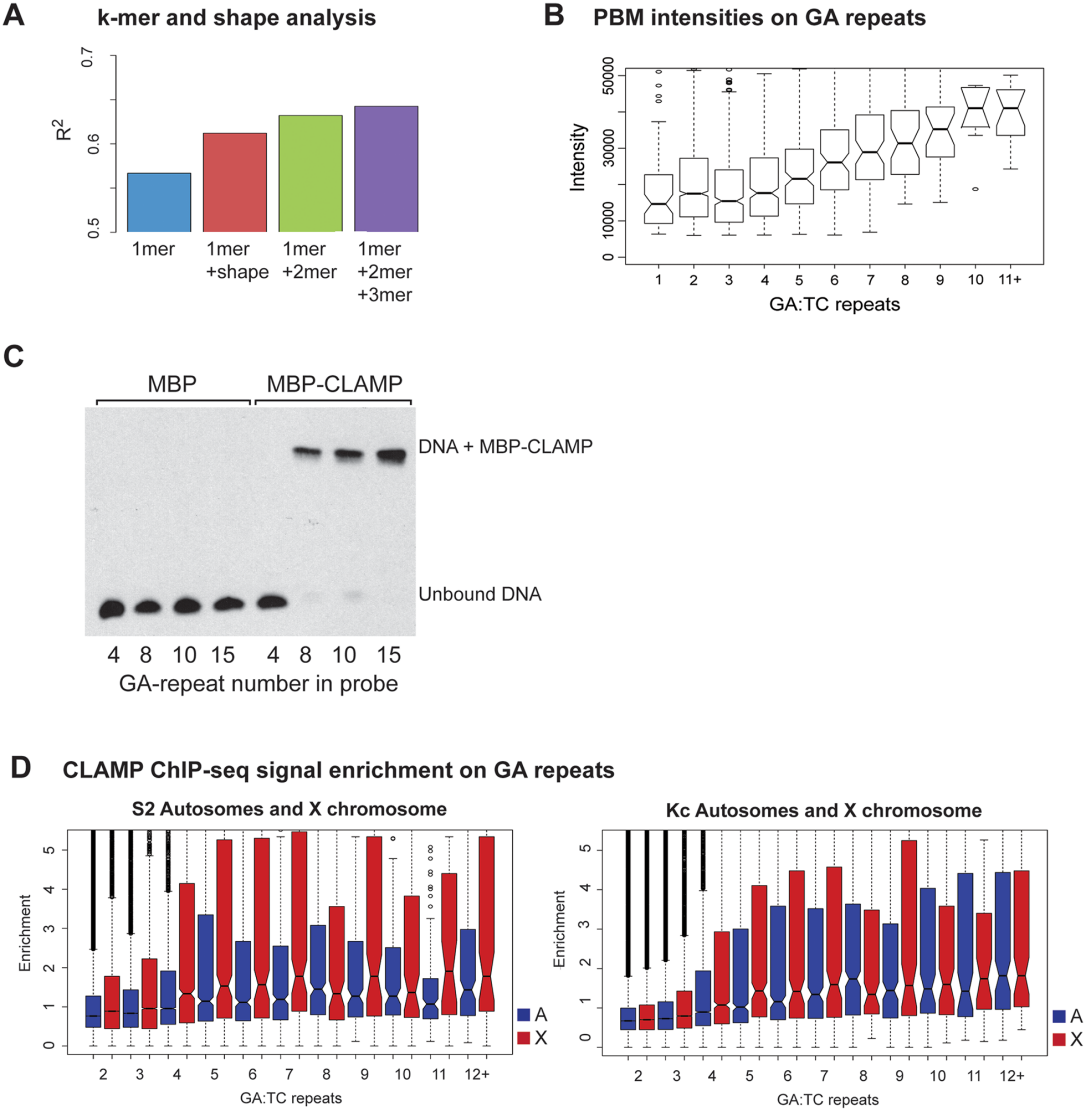
Increasing the number of GA-dinucleotide repeats increases CLAMP occupancy. **A)** A multiple linear regression to test contribution of sequence length (k-mer) and shape to overall binding. Adding dinucleotide (2mer) features to the sequence-only (1mer) model increases the performance more than adding DNA shape features, indicating the importance of dinucleotides in CLAMP-DNA recognition. Adding trinucleotide (3mer) features further increases the performance marginally. **B)** CLAMP PBM binding for GA-dinucleotide repeats of different lengths is plotted as box plot distributions. The y-axis is the PBM intensity score for each number of GA-repeats, which are plotted along the x-axis, e.g. 1 = GA, 2 = GAGA. **C)** An electrophoretic mobility shift assay to test MBP-CLAMP binding to increasing numbers of GA-repeats. The labeled probes contain GA-repeats of 4 (8-bp), 8 (16-bp), 10 (20-bp) and 15 (30-bp) centered within a 60-bp sequence. The first four lanes are reactions containing MBP control protein with DNA, and the next four are MBP-CLAMP with DNA. **D)** Input-normalized CLAMP ChIP-seq signal enrichments at GA-repeats of different lengths are given for the X-chromosome (red) and autosomes (blue) from male S2 (top) and female Kc cells (bottom). The x-axis shows the number of GA-repeats e.g. 1 = GA, 2 = GAGA.

Next, we used a statistical machine learning approach called L2-regularized multiple linear regression (MLR)[27,31] to determine features that were the best predictors of *in vitro* CLAMP binding based on gcPBM data. We found that the addition of dinucleotides to the model (1mer+2mer) was a better predictor of CLAMP binding than the addition of DNA shape parameters (1mer+shape) (p<0.001) (Fig 3A). The addition of trinucleotides (3-mers) did not substantially further increase the ability to predict binding (1.6% percent performance gain). Therefore, we concluded that dinucleotides were the best predictors of CLAMP binding *in vitro*.

Because GA-dinucleotides were the best predictors of CLAMP binding, we examined the binding of CLAMP to PBM probes containing increasing lengths of GA-repeats directly. We found that there is a stepwise increase in binding to longer repeats that begins between 4 repeats (8-bp) and 5 repeats (10-bp) corresponding with extending the motif beyond the 8-bp core of the motif (Fig 3B). The increase in binding plateaus at 10 repeats (20-bp) and there are very few probes that have more than 11 repeats within the 36-bp probe sequence so they were binned together (Fig 3B, number of probes for each repeat length are reported as S6 Table).

In order to ask whether the signal on the PBM increases with the number of GA-repeats because a single CLAMP protein molecule binds to DNA with greater affinity or multiple CLAMP molecules bind to a single probe, we performed EMSAs with GA-repeat probes. Again, we are using MBP-tagged CLAMP to test if binding increases with GA-length independent of potential dimerization of the GST-tagged CLAMP. The probes contained 4, 8, 10, and 15 GA-repeats (8-bp, 16-bp, 20-bp, 30-bp) embedded within a 60-bp sequence. When MBP-CLAMP was added to each labeled probe, there was an increase in the shifted, protein-bound DNA signal for all probes except the 8-bp probe that corresponds only to the core CLAMP motif (Fig 3C). There was a single shifted species for all probes, even the 30-bp GA-repeat which indicates that increased signal is likely due to the greater frequency of a single CLAMP protein binding to DNA not multimerization of the CLAMP DNA binding domain on probes with longer repeats. We cannot fully exclude the possibility that GST-CLAMP dimerizes in the gcPBM experiments, but by testing CLAMP binding with a non-dimerizing tag we observe the same trends in binding indicating it is likely CLAMP rather than the tag predominantly influences binding to longer GA-repeats.

Consistent with the *in vitro* analysis, *in vivo* occupancy of CLAMP from ChIP-seq data in both male (S2) and female (Kc) cells increases as the number of GA-repeats increases on both the X-chromosome (red) and autosomes (blue) (Fig 3D, number of repeats analyzed in the genome reported in S7 Table). We also compared binding on individual autosomal arms for each different GA-repeat length and observed similar trends (S2 cells: S4A Fig and Kc cells: S4B Fig). As previously reported, *in vivo* CLAMP binding is modestly increased on the X-chromosome in S2 cells (male) when compared to Kc cells (female) and autosomes due to synergistic interactions between MSL complex and CLAMP (Fig 3D)[20]. Overall, CLAMP occupancy increases both *in vivo* and *in vitro* as the number of GA-repeats increases on both the X-chromosome and autosomes.

### The X-chromosome has increased density of GA-repeats and CLAMP occupancy compared to autosomes

**Fig 4:**
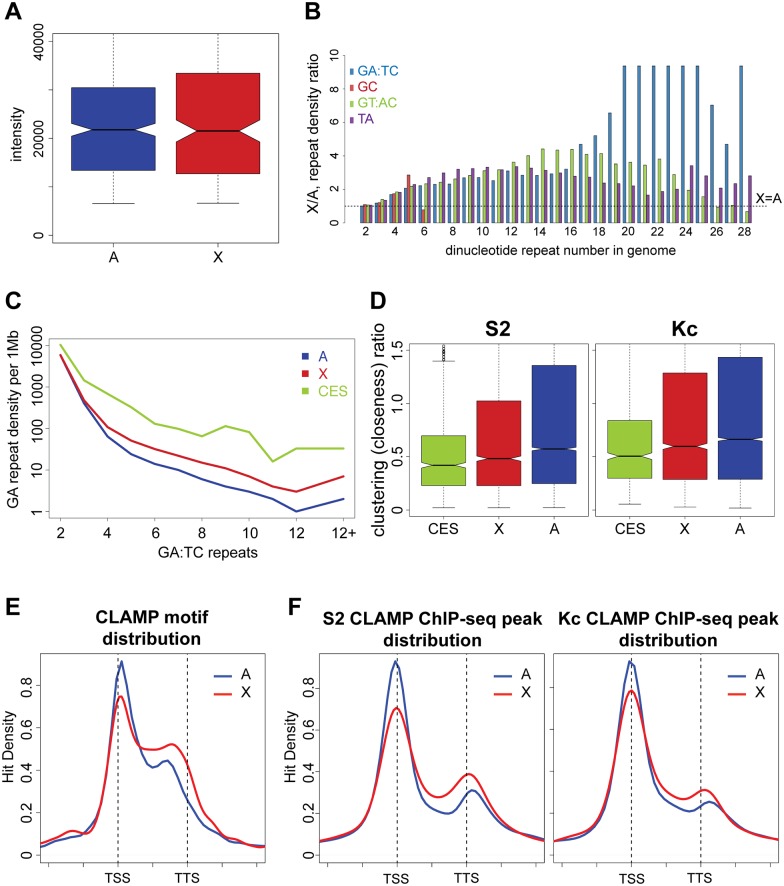
The X-chromosome has more GA-repeats than autosomes and the CES have clustering of CLAMP occupancy. A) Distribution of the PBM intensities of the probes aligned to the X-chromosome (red) versus autosomes (blue). B) The density of different dinucleotide repeats on X-chromosome is compared with autosomes for different repeat lengths within the *D*. *melanogaster* genome. Any value above 1 indicates a higher repeat density on X-chromosome compared with autosomes. C) Density of GA repeats (1 = GA, 2 = GAGA, etc.) per 1 Mb along Autosomes (A, blue), the X-chromosome (X, red), and X-chromosome CES (CES, green). D) Top: Average distance between n neighboring CLAMP ChIP-seq peaks in S2 (left) and Kc (right) cells. Peaks are separated as within CES (green), on X (red), or on autosomes (blue). Bottom: The average distances are replotted for S2 (left) and Kc (right) cells after normalization using distances measured between n random peaks. E) A histogram showing the frequency of each 15-bp CLAMP motif at a particular location relative to genes on the X (red) or autosomes (blue). The profiles are shown around gene bodies and the range is from one gene length before the TSS to one gene length after the TTS. The distance from the TSS is normalized to gene length for each gene. F) Average gene profiles of CLAMP ChIP-seq peaks on X (red) and autosomes (blue) from S2 cells (male) and Kc cells (female). Normalization was conducted as for E.

While CLAMP is necessary for MSL complex recruitment specifically to the X-chromosome, CLAMP occupies GA-rich binding sites all over the genome [20]. In fact, the average *in vitro* binding to the group of X-linked sequences on the PBM does not differ significantly from the average binding to autosomal sequences (Fig 4A). There is a statistically significant increase in CLAMP occupancy at X-linked sites only in the top quantile of binding affinity (p-value <0.01) (S5A Fig). These *in vitro* data are consistent with the binding of CLAMP to similar GA-rich motifs throughout the genome based on ChIP-seq analysis [20]. Therefore, we hypothesized that rather than enhanced CLAMP binding to each individual site, an increased density of GA-repeats may occur on the X-chromosome that would generate more CLAMP binding sites and clustering of groups of CLAMP sites in closer proximity to each other.

The CLAMP binding sites we identified from our custom PBMs are 2.2 fold X-enriched, a slightly greater enrichment than the 1.8 fold enrichment of the MRE sequences [13]. Because CLAMP binds more strongly to sites containing more GA-repeats (Fig 3), we compared the GA-repeat content of the X-chromosome to autosomes. Classical FISH studies conducted before the sequencing of the *Drosophila* genome demonstrated that many types of dinucleotide repeats are increased on the X-chromosome[8]. However, this previous work did not precisely define the dinucleotide repeat content of the X-chromosome vs. autosomes, so we computationally measured the ratio of dinucleotide repeats of all lengths throughout the genome. We determined that the density of all types of dinucleotide repeats is strongly increased on the X-chromosome vs. autosomes (Fig 4B). We also compared X to individual autosomal arms (and observed the same trends (S5B Fig, S8 Table). Interestingly, long GA-repeats are more highly enriched on the X-chromosome than other types of dinucleotide repeats, consistent with the X-enrichment of long GA-rich CLAMP binding sites (Fig 4B). The regions of highest MSL complex occupancy (CES, green) are even more enriched for GA-repeats than other regions of the X-chromosome (red) or autosomes (blue) (Fig 4C). Therefore, we hypothesized that increased density of long GA-repeats within CES increases the density of CLAMP binding sites, which then promotes MSL complex targeting to CES.

To test this hypothesis, we directly measured the average distances between *in vivo* CLAMP-occupied sites at CES, other sites on X, and autosomal sites and compared them to the values expected for random site distribution (Fig 4D, S6 Fig, p-values reported as S9, S10, S12 and S13 Tables). Specifically, we measured the distances between CLAMP ChIP-seq peaks in S2 and Kc cells and calculated average distance of each site to the closest 1–4 neighboring sites (number of peaks used are reported as S11 Table). The CES have smaller distances between CLAMP sites than all other loci on the X-chromosome or autosomes, including individual sites on autosomal arms (S6A Fig). To account for difference in absolute counts of the CLAMP sites within CES and other regions, we normalized our data by comparing with a random distribution of the same number of sites. We demonstrated that CLAMP sites are relatively closer to each other around the CES compared with all other genomic regions in the presence (S2 cells) and the absence of MSL complex (Kc cells). Therefore, the clustering of CLAMP peaks is increased around CES even when normalized for increased peak number and is independent of MSL complex. Overall, these data support our hypothesis that CES can be distinguished from other genomic locations prior to MSL complex recruitment due to their increased density of CLAMP sites.

Unlike most transcriptional regulators that localize to transcription start sites (TSS), the MSL complex is recruited to active gene bodies and transcription termination sites (TTS) to promote the progression of RNA Polymerase II across genes [10,11,32,33]. Therefore, we hypothesized that increased density of CLAMP peaks and motifs over gene bodies and 3’ UTRs on the X-chromosome vs. autosomes would cause CLAMP occupancy to be more broadly distributed on X-linked genes increasing overlap with MSL complex binding sites. To test this hypothesis, we mapped the position of the CLAMP binding motif from our top bound sites to the genome (Fig 1A: IV) and generated an average gene profile to measure the frequency of its distribution across genes (Fig 4E). Next, we compared the location of CLAMP motifs to a similar plot of CLAMP occupancy from ChIP-seq data on the X-chromosome and autosomes in both male (S2) and female (Kc) cells (Fig 4F). We also quantified the motif density and CLAMP occupancy ratios of the gene body (from TSS+250 bp to the TTS) to the 5’ end (TSS+/- 250 bp) for the X-chromosome, autosomes, and individual autosome arms (S7A and S7B Fig). CLAMP motifs and occupancy are concentrated at the TSS and TTS of genes on both the X-chromosomes and autosomes. However, on the X-chromosome, CLAMP motifs and occupancy levels are more broadly distributed over gene bodies relative to TSS compared with than on autosomes. Overall, the localization of CLAMP on the X-chromosome is more biased towards gene bodies and the TTS and away from the TSS than on autosomes. Therefore, X-linked CLAMP binding sites have a greater chance of co-occupying sites where MSL complex binding would be stabilized by gene body chromatin marks with known roles in dosage compensation such as H3K36me3 [34] than autosomal sites.

### The CLAMP protein and X-enrichment of its GA-repeat binding sites are conserved across species

**Fig 5:**
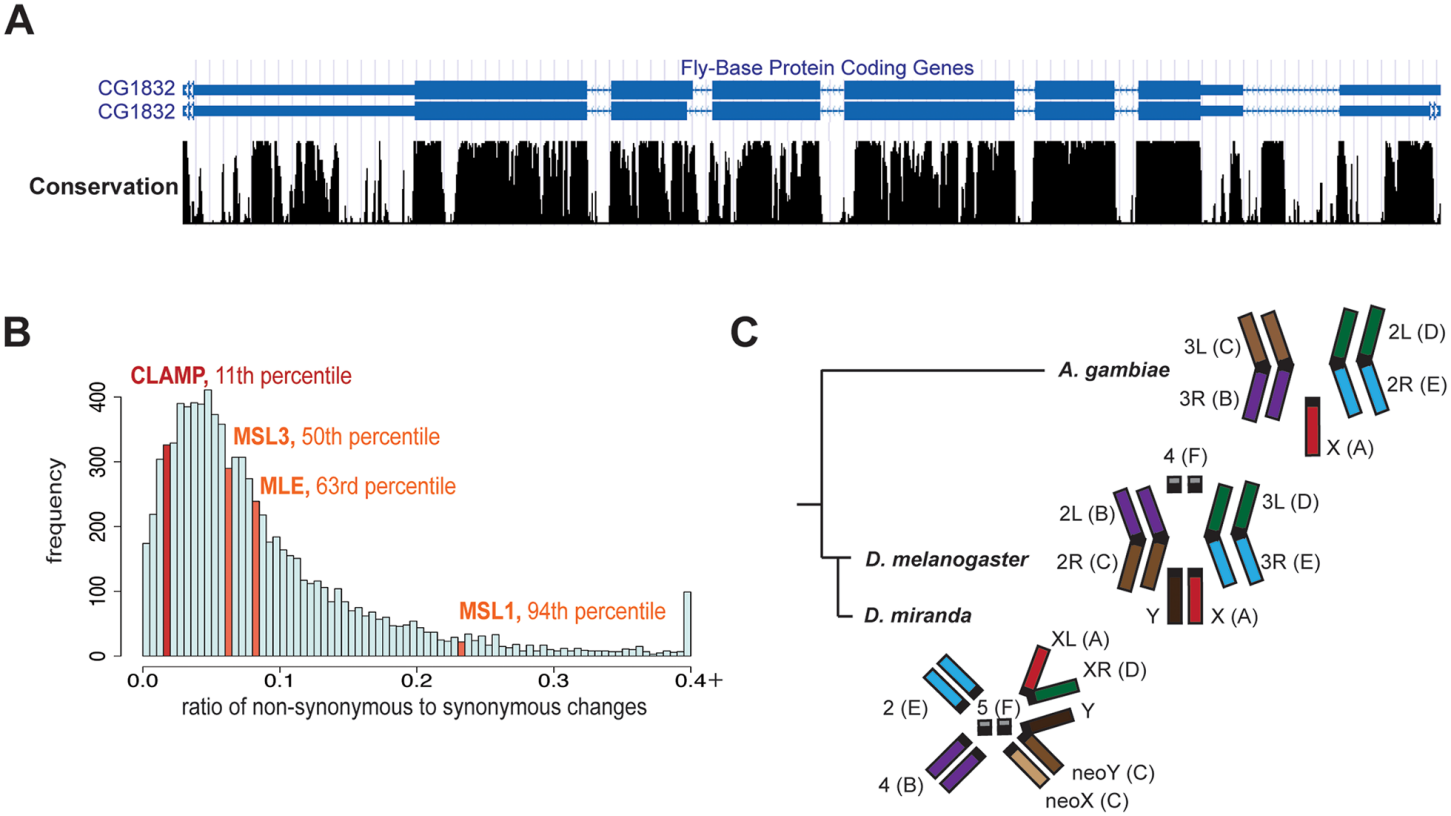
CLAMP and enrichment of the CLAMP motif are highly conserved across species. **A)** A schematic of the CLAMP (CG1832) gene from *D*. *melanogaster* is shown in blue. Conservation of the CLAMP gene sequence compared to orthologues in other *Drosophilids* and the mosquito (*A*. *gambiae*) is shown below (UCSC Genome Browser). **B)** The genomic distribution of the ratio of non-synonymous to synonymous changes is given (dN/dS ratio) for all 8560 genes with 1:1 orthologues between *D*. *melanogaster* and *D*. *simulans*. Percentiles are listed for CLAMP and the MSL components for which 1:1 orthologues were present: MSL1, MSL3 and MLE. **C)** Schematic of male *A*. *gambiae*, *D*. *melanogaster*, and *D*. *miranda* chromosomes and corresponding Muller element letters. Colors are matched based on Muller element identity[6] [62].

Recent work demonstrated that dosage compensation across *Diptera* evolved independently as many different chromosome arms transitioned from autosomal to X- chromosome identities [6]. To determine whether CLAMP could be a key factor involved in the evolution of the X-chromosome across *Diptera*, we first examined its conservation. The CLAMP gene is highly conserved across all *Diptera* that have sufficient sequencing data to find orthologs (Fig 5A, S8 Fig). Moreover, the DNA binding domain (a.a. 350–561) always has six tandem zinc fingers and is very highly conserved across diverse species (S14 Table) [35]. The glutamine-rich domain is less conserved in terms of specific amino acid position but its glutamine-rich nature is conserved across species (S9 Fig, S14 Table) [36].

To determine how conserved the gene encoding CLAMP is compared to all other genes in *Drosophila*, we compared the ratio of non-synonymous to synonymous changes in CLAMP between *D*. *melanogaster* and *D*. *simulans* to the same ratio for all 1:1 orthologs in the *D*. *melanogaster* species group [37] (Fig 5B). Among *Drosophilids*, CLAMP is more highly conserved than all but 11% of *Drosophila* protein coding sequences. Moreover, CLAMP is much more highly conserved than the MSL complex components for which 1:1 orthologue data were available: MSL1, MSL3 and MLE (maleless). The low level of conservation of MSL components is consistent with previous measurements [38]. Therefore, it is possible that the dosage compensation machinery could have co-opted the function of the more ancient CLAMP protein independently in multiple species as X-chromosomes evolved.

**Fig 6:**
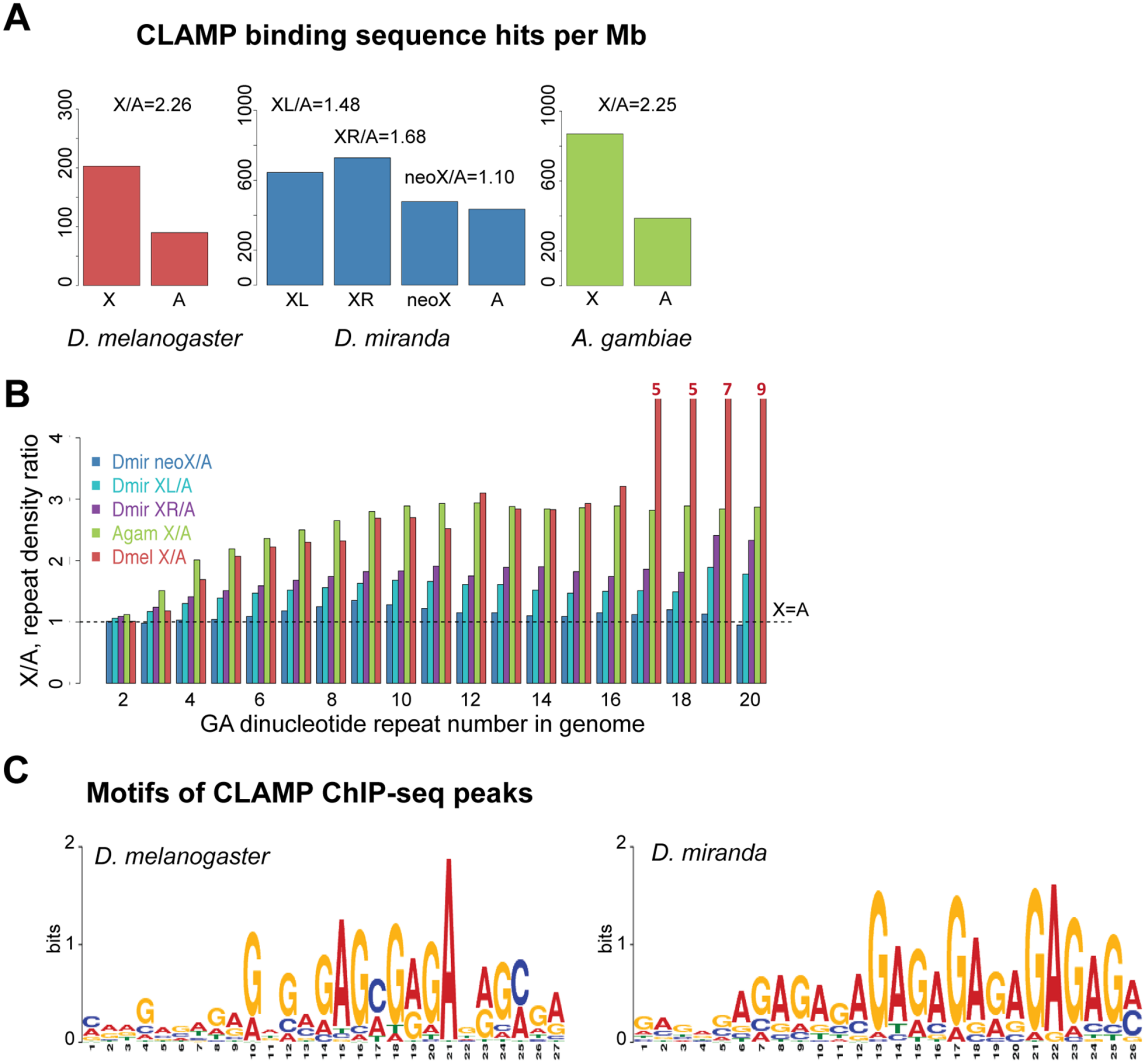
CLAMP binds to similar *in vivo* X-enriched binding sites in *D*. *miranda* and *D*. *melanogaster*. **A)** The ratio of *in vitro* CLAMP binding site density (number of CLAMP binding sequence hits per Mb) for X versus autosomes is plotted for *D*. *miranda*, *D*. *melanogaster* and *A*. *gambiae*. Autosomes of *D*. *melanogaster* are chromosomes 2L (Muller-B), 2R (Muller-C), 3L (Muller-D), 3R (Muller-E) and 4 (Muller-F); autosomes of *D*.*miranda* are chromosomes 2 (Muller-E), 4 (Muller-B) and 5 (Muller-F); and autosomes of *A*. *gambiae* are chromosomes 2L (Muller-D), 2R (Muller-E), 3L (Muller-C), and 3R (Muller-B). **B)** The density ratios of GA-repeats on individual X-chromosome(s) vs. autosomes for different repeat lengths in the *D*. *melanogaster*, *D*. *miranda* and *A*. *gambiae* genomes are plotted. Any value above 1 indicates a higher repeat density on the X-chromosome compared to autosomes. **C)** CLAMP ChIP-seq motifs are shown for *D*. *melanogaster* and *D*. *Miranda* larval ChIP-seq data. Motifs are found using MEME-ChIP for the peak regions (peak centers +/-100bp).

Based on the strong conservation of CLAMP across species, we hypothesized that the CLAMP motif may have become X-enriched independently on different X-chromosomes in multiple species. To test this hypothesis, we compared the density of CLAMP motifs on the X-chromosome vs. autosomes in several *Diptera* in which appropriate assemblies and sequence information defining the X-chromosome is available. Among *Drosophilids*, we compared *D*. *melanogaster* with the distantly related *D*. *miranda* (30 million years apart) [39] that has both ancient X-chromosomes (XL,XR) and a newly evolving X-chromosome (neoX) (Fig 5C). Therefore, it is possible to examine the neoX as an intermediate in the evolution of dosage compensation [14,16,17]. As noted previously, the density of CLAMP binding sequences per megabase on the X-chromosome is 2.2-fold enriched compared with autosomes (Fig 6A: red, S15 Table). Within *D*. *miranda*, the ancient XL and XR chromosomes are more enriched for CLAMP motifs compared with the newly evolving neoX chromosome that has a modest 1.1 fold enrichment (Fig 6A: blue, S15 Table).

To determine the degree of conservation of the X-enrichment of the CLAMP motif outside of *Drosophilids*, we examined the X-enrichment of the CLAMP motifs in *Anopheles gambiae*, the distantly related mosquito (250 million years apart) with a fully assembled genome. For measurements of motifs and repeat density, we found that fully assembled genomes and scaffolds did not correlate well, and therefore we used only fully assembled genomes. Interestingly, we observe the same X-enrichment of CLAMP motifs in *A*. *gambiae* and *D*. *melanogaster* (2.2-fold) revealing conservation across 250 million years of evolutionary time (Fig 6A: green). Because CLAMP binds more strongly to long GA-repeats (Fig 3), we determined the density of GA-repeats of different lengths across species. We observed elevated GA-repeat density on the X-chromosome in *A*. *gambiae* and the ancient X-chromosomes in *D*. *miranda* (XL and XR) (Fig 6B). In contrast, the newly evolving neo X-chromosome has very modest enrichment of GA-repeats compared with autosomes (Fig 6B). We also analyzed other types of dinucleotide repeats and found that they are also X-enriched that suggests a general expansion of dinucleotide repeats (S10 Fig and S16 Table, *D*. *miranda*, S17 Table, *A*. *gambiae*). However, CLAMP recognizes only a motif containing GA-repeats [20] and therefore it is unlikely that the expansion of the other repeats would alter CLAMP occupancy. To test that our combined autosome scores were representative of individual autosomal elements, we compared the density ratios to each individual arm, and in all cases dinucleotide repeats were enriched on X with the exception of Muller Element F that is highly heterochromatic (S11 Fig, *D*. *miranda*, and S12 Fig, *A*. *gambiae*). Therefore, the X-enrichment of GA and other dinucleotide repeats is conserved across 250 million years of evolution and is increased on ancient X-chromosomes when compared with a newly evolving X-chromosome.

To determine whether CLAMP binds to the same motif across species, we performed ChIP-seq in *D*. *miranda* larvae and compared our motifs with those from *D*. *melanogaster* larvae. Overall, the motifs that we derived from *D*. *miranda* larvae ChIP-seq are similar to those from *D*. *melanogaster* larvae, *in vivo* motifs from tissue culture cells and the *in vitro* gcPBM motif (Figs 6C and 1A). When we compared the genomic sequences of each *D*. *miranda* chromosome to the respective motif identified from *D*. *miranda*, we found that ancient XL and XR had the strongest motifs with the lowest p-values (S13A Fig). Furthermore, when we obtained motifs from the peaks bound at the highest level (top 1000 peaks), we observed even more dramatic elongation of GA-repeats within the *D*. *miranda* motif (S13B Fig). Next, we performed controls to assure the specificity of our motifs by examining the occurrence of the true motifs within 1 kb genomic regions surrounding ChIP-seq peaks vs. scrambled and randomized control genomic regions (S13C Fig). As p-values decreased, the specificity of the motifs increased, validating the motifs. Furthermore, the motifs that we obtained were X-enriched and their X-enrichment increased as the p-values decreased (S13D Fig, S15 Table). Overall, the CLAMP protein interacts with a very similar sequence across 30 million years of evolutionary time suggesting that it is an ancient factor that recognizes GA-repeats.

Although the *D*. *melanogaster* and *D*. *miranda* CLAMP *in vivo* motifs are very similar (Fig 6C), we observed one intriguing difference: The incidence of a cytosine instead of an adenine at position 4 within the GA-rich core occurs more often in *D*. *melanogaster* (14.3%) than in *D*. *miranda* (6.0%). While the interaction of CLAMP with DNA is highly sequence-specific [20], the MSL2 component of MSL complex has some specificity for interacting with MREs via its CXC domain when expressed at high levels *in vitro* [18]. This specificity is conferred by interaction between the MSL2 R543 residue and the cytosine that can occur within some MRE motifs at position 4[18]. Therefore, we examined the *D*. *miranda* MSL2 CXC domain and determined that it lacks 4 out of 9 cysteine residues that are required to maintain its structure and the key R543 residue that contacts the cytosine specifically (S14 Fig)[40]. Therefore, differences in DNA binding specificity of CLAMP correlate with changes in the MSL2 protein that alter its DNA binding specificity, suggesting potential co-evolution of these two proteins.

In summary, we have integrated biochemical, *in vivo*, genomic, and evolutionary approaches to support a model by which expansion of GA simple sequence repeats on the X-chromosome promotes increased density of CLAMP within CES which then targets MSL complex. It is likely that increased density of the maternally loaded CLAMP protein at CES functions together with previously identified regulators such as the *roX* RNAs [41] and the H3K36me3 histone mark [34,42] to promote specific recruitment of MSL complex to the X-chromosome.

## Discussion

Upon the evolution of heterogametic species, the process of dosage compensation became essential to ensure the appropriate balance of gene expression between males and females and the X and autosomes. Distinguishing the X-chromosome from autosomes is the key step in this process because MSL complex must be targeted to the correct chromosome to ensure the fidelity of dosage compensation. Here, we demonstrate that in several species this process likely involved enriching the evolving X-chromosomes for long GA-repeat binding sites that can be recognized by the highly conserved CLAMP protein that recruits MSL complex.

CLAMP binding sites are not X-specific as the CLAMP protein binds to similar GA-rich sequences all over the genome [20]. We propose that a higher density of sites within CES that contain longer GA-repeats evolved to optimize CLAMP binding on X to better target MSL complex for dosage compensation. Then, it is likely that the increased density of CLAMP at CES functions together with other cofactors with known roles in MSL complex recruitment such as H3K36me3 [34,42] and *roX* RNAs [41]. Once this initial process of X-chromosome identification occurs, synergistic interactions between maternally loaded CLAMP and the MSL complex [20] increase the X-enrichment of both factors.

Interestingly, the CLAMP motif is much longer than most transcription factor binding sites. It is possible that the length of the CLAMP binding site ensures specificity by reducing the promiscuity of its binding and allowing it to compete with other similar proteins. In addition, recent work on transcriptional regulators in budding yeast has implicated the sequence context of transcription factor binding sites outside of the core binding site as critical for the recognition process {Levo, 2015 #414]. Therefore, current approaches to identifying transcription factor binding site motifs have likely underestimated their length due to the approaches used that often allow detection of only short motifs. In the future, it will be important to determine transcription factor recognition motifs using approaches like gcPBM that uses *in vivo* sequences to identify direct binding site motifs.

There are several mechanisms by which the GA-repeat number could have been increased including expansions due to slippage of DNA polymerase. Helitron transposons containing GA-rich sequences have also been implicated in the X-enrichment of these sequences in *D*. *miranda* {Ellison, 2013 #377}. It is possible that expansions of GA dinucleotides occurred within these transposons after they landed on the X-chromosome. These GA-repeat expansions could have been further propagated by gene conversion events that also occurred during the evolution of dosage compensation [17]. Finally, long repeat sequences such as the 1.688 elements that produce siRNAs function during dosage compensation via an unknown mechanism [43]. Therefore, it is possible that GA-repeat elements have been expanded over evolutionary time because of a general role in promoting dosage compensation. To support this hypothesis, a recent report identified GA-rich binding motifs almost identical to those that we characterized as CLAMP binding sites within the strongest MSL complex binding sites in three additional *Drosophila* species [44].

Motifs that contain GA-repeats have been implicated in diverse processes that all involve generating open chromatin regions. GA-repeat containing motifs are highly enriched at sites that promote pausing of RNA Polymerase II and at developmentally regulated DNase I hypersensitivity sites [45,46]. Furthermore, a GA-repeat motif is one of the two motifs that are enriched at genes that are activated first during the maternal to zygotic transition [47]. The well-studied GAGA factor (GAF) protein also recognizes similar sequences to the CLAMP protein and has been implicated in pausing of RNA Polymerase II and opening of chromatin [48]. Overall, it is likely that the dosage compensation machinery has evolved to take advantage of targeting GA-repeats that mark open chromatin regions to ensure that it only identifies active genes for further transcriptional upregulation by the MSL complex.

It is possible that GA-rich sequences have roles in dosage compensation outside of *Diptera*. For example, it has been proposed that upregulation of the single active X occurs in mammals and this process is mediated by targeting the conserved MOF histone acetyltransferase component of MSL complex [49]. Moreover, GA-repeats were found to be significantly enriched within regions of the X-chromosome that escape X-inactivation (X escape regions) [50]. There are no strong homologues of CLAMP in mammals but there are several possible functional orthologs such as the ETS family transcription factor GABP1 (GA binding protein-1) [51]. Furthermore, in *C*. *elegans*, there is an early upregulation of both X-chromosomes that is also mediated by the MOF histone acetyltransferase [49]. One of the zinc finger proteins that targets the *C*. *elegans* dosage compensation machinery is SCC-2 (sister chromatid cohesion—2) which recognizes a GA-repeat sequence very similar to the CLAMP binding motif [52]. Therefore, it is possible that GA-repeats are involved in dosage compensation beyond *Diptera* and this will be an exciting area for future investigation.

## Materials and Methods

### gcPBM design, experimental methods, and data analysis

#### Design of custom genomic-context Protein-Binding Microarray (gcPBM)

PBM probes were designed to assay three classes of DNA sequences defined by (1) the presence of an MRE sequence (derived from MSL3-TAP ChIP-seq data [13]), and (2) *in vivo* binding determined by CLAMP ChIP-seq experiment[20]. CLAMP ChIP-seq peaks (identified at a false-discovery rate of 0.001) [13] were examined for the presence of CLAMP binding sites [20]. Matches to two previously identified minimal *in vitro* CLAMP binding motifs from universal PBM (uPBM) experiments[20] were used to define a CLAMP binding site (CLAMP motif, cutoff <7.8; CLAMP secondary motif, cutoff < 6.0). CLAMP ChIP-positive regions, each containing a minimal CLAMP *in vitro* binding site, were then divided based on the presence or absence of an MRE sequence and labeled as ChIP+/MRE+ or ChIP+/MRE-, respectively. Similarity to the MRE motif [13] was determined using the FIMO tool [53]. Sequences with similarity of less than p-value = 10^−4^ were labeled as MRE+, and sequences with similarity of higher than p-value = 10^−3^ were labeled as MRE-. A third set of probes focused on promoter regions from the fly genome (500 bp up and downstream of transcription start site; fly genome build dm3 from UCSC) that did not overlap with CLAMP ChIP-positive regions (i.e., contain a CLAMP binding site but were not bound *in vivo*). Promoter regions were ranked according to CLAMP binding motif scores (as described), for the top-scoring 250 promoter regions, PBM probes were constructed for all identified CLAMP binding sites, and are referred to as ChIP-/MRE+. We also tiled the genomic regions including the *roX1*, *roX2* and CES5C2 MSL complex binding sites. Our design protocol resulted in 3944 ChIP+/MRE+ probes, 2313 ChIP+/MRE- probes, and for 1282 ChIP-/MRE+ probes. All probes on the PBM are 60-nucleotides (nt) long and contain a 24-nt primer sequence (positions 37 to 60) and a 36-nt variable region. Probes were constructed with the minimal CLAMP binding site from uPBMs centered at nucleotide position 19, allowing 17-nt of genomic flanking DNA on either side. All probes are included four times on the array in each orientation (i.e., forward sense and reverse complement), resulting in eight replicate measurements per unique binding sequence. See S1 Table for complete design details for the gcPBM.

Probes were defined as PBM+/- based on their PBM signal intensities. Probe intensities had a bimodal distribution (Fig 1C). We selected a value of 6500, which is between the two peaks of the distribution, as the threshold to define binding *in vitro*: probes with an intensity higher than 6500 were labeled as PBM+ (“bound”) and probes with an intensity lower than 6500 were labeled as PBM- (“not bound”). gcPBM data will publically available upon publication:

#### *In vitro* protein expression for protein binding microarrays (PBMs)

The pDEST15 GST expressing Gateway vector (Invitrogen) was used to generate the expression plasmid for the C-terminal CLAMP four and six zinc finger constructs. For the six-finger construct (amino acid residues 350–561), the following primers were used for cloning: forward primer 5’-ACTGTTAAAACTGAAAATCCGTCCAA-3’ and reverse primer 5’-CTATAACCCACCGATAATCACTTCCTGT-3’. For the four-finger construct, (amino acid residues 412–561), the following primers were used for cloning: 5'-GGCGAGAAACCCTATAAATGCCAAAC-3' and reverse primer 5’-CTATAACCCACCGATAATCACTTCCTGT-3’. Cloning was performed using standard Gateway Technology (Invitrogen). Proteins were expressed using the TnT Quick-coupled Transcription and Translation Systems Kit (Promega). *In vitro* transcription and translation reactions were carried out according to standard protocols with the following adaptation: zinc acetate was added to a final concentration of 50 μM.

Proteins were quantified using Western blotting with a GST dilution series as a standard. The concentrations of the proteins used for PBMs were as follows: 1) C-terminal 4 zinc finger protein (24.3 nM); 2) C-terminal 6 zinc finger protein (13.9 nM).

#### PBM experiments and initial analysis

PBM experiments were performed using a custom-designed oligonucleotide array (Agilent Technologies, Inc., AMADID #037964, design described above). The array was converted to a double-stranded DNA array by primer extension and used in PBM experiments essentially as described previously[22,54]. Protein samples containing C-terminal 4- and 6-finger fusions of the CLAMP protein to GST (preparation as described above) were incubated in four different chambers of a gcPBM microarray at a final concentration of ~ 200 nM for 1 hour in binding buffer (10 mM Tris-HCl pH 7.4; 0.2 μg/ul bovine serum albumin (BSA) (New England BioLabs #B9001S); 0.3 ng/ul salmon testes DNA (Sigma, #D7656); 2% non-fat dry milk (Stop & Shop brand); 1 mM dithiothreitol (DTT); 1 mM ethylenediaminetetraacetic acid (EDTA); 50 μM zinc acetate dehydrate; 80 mM NaCl). Protein-bound arrays were then washed and incubated with antibody for 20 min (0.05 mg/ml Alexa 488-conjugated anti-GST antibody (Invitrogen, #A11131). Microarray scanning, quantification, and data normalization were performed using GenePix Pro ver. 6 (Axon) and masliner (MicroArray LINEar Regression) software[55] as previously described[22,54]. For all probe sequences, median fluorescence intensities were determined for the eight replicate probes. PBM data are available at the with the following NCBI GEO accession number: GSE83442.

#### Motif analysis for both PBM and ChIP-seq data

Motif analysis was performed using the MEME software suite: specifically, MEME[56] was used for the motif from PBM data and MEME-ChIP[57] was used for the motifs analysis from CLAMP ChIP-seq data. The parameters were set to allow one motif occurrence per input sequence; both forward and reverse sequence orientations were considered; minimal motif length was set to 12 bp; other parameters were set to default values. Motif detection at the ChIP-seq peaks as well as in random and scrambled sequences was performed using the FIMO tool[53] with default parameters. Motif logos were created using Weblogo[58].

### DNA shape analysis

The distribution of the logarithmic signal intensity of the PBM probes indicated two mixed Gaussian distributions, with the Gaussian distribution of higher mean likely representing CLAMP binding. Mixture Gaussian modeling was used to separate out strongly bound probes. First, a two-Gaussian model was fitted to the logarithmic signal intensity of the probes. Then probes that were assigned to the higher Gaussian with confidence of ≥ 0.95 were defined as strongly bound probes. Probes that ambiguously mapped to the *Drosophila* genome were removed. The remaining probes were then fed as input to the MEME suite for motif discovery[59] using default settings. Following this step, the probes were aligned according to the motif generated by MEME.

Using the aligned PBM probes, L2-regularized multiple linear regression (MLR) was used to model the DNA binding specificity of CLAMP. Mononucleotide (1mer), dinucleotide (2mer), and trinucleotide (3mer) features were extracted from the sequences, and the four DNA shape features minor groove width, propeller twist, roll, and helix twist were derived from our DNA shape method[60]. These features were encoded for different models as previously described[31]. These features were then used as the predictor variables and the signal intensity of the probes as the response variable for the L2-regularized MLR. The coefficient of determination *R*^*2*^ between predicted and experimental probe intensities was calculated as the performance measure of the models using 10-fold cross validation.

### Protein expression, DNA labeling, and methods for EMSA

#### Protein expression from E.coli for EMSAs

The pMAL-c5X Vector containing the MBP (Maltose Binding Tag) was used to express MBP fusion CLAMP 6 zinc finger protein using the Nde1 and Sbf1 restriction enzyme sites. For large-scale E. coli expression, the CLAMP cDNA was codon optimized and ordered (Life Technologies). Additionally, for optimal expression amino acids after the zinc fingers that had low-predicted structure (a.a. 528–561) were not include in these constructs. The following primers were used for cloning the 6 zinc finger domain of CLAMP: Forward primer 5’-TACGATCATATGTCCGGTAGCGTTAAACAGAGTGTTACCGT-3’ and reverse primer 5’-TACGATCCTGCAGGCTATTACAGACCGCCAATAATAACTTCTT-3’ TTACAGACCGCCAATAATAACTTCTT 5’. The pMAL protein fusion and purification system from NEB (Catalog # E8200S) was used.

CLAMP protein was overexpressed in strain *E*. *coli* Star (Invitrogen) in LB media containing 50 μg/ml ampicillin and 90 μM ZnCl_2_. Cultures were grown to mid-log phase at 310 K and expression was induced with 0.4 M IPTG. The cells were then further grown for 18 hours at 293 K and harvested by centrifugation. Cells were lysed by sonication on ice in a buffer containing 20mM Tris pH 7.5, 90 μM ZnCl_2_, 1 mM MgCl_2_ and 90mM KCl. The soluble lysate was then loaded onto an amylose column. Unbound protein was removed with a wash buffer containing 20 mM Tris pH 8.0, 100 mM NaCl, and 90 μM ZnCl_2_. MBP-CLAMP was then eluted in the same buffer containing 10 mM Maltose. 5% glycerol was added and protein aliquots were stored at 193 K.

#### DNA labeling for electrophoretic mobility shift assay

The CLAMP binding sequences were end labeled with Biotin using Biotin 3’End Labeling Kit (Thermo Scientific Pierce Cat#89818) with the following adaptations. Each strand of DNA was Biotin labeled independently then annealed by heating to 95°C for 10 mins then removing from heat to slowly cool to room temperature (20°C). Single strand DNA digestion was performed to remove un-annealed DNA for 30 mins at 37°C with Mung Bean Nuclease 2,000 U/uL (New England Biolabs Cat#M0250S) followed by Chloroform:isoamyl alcohol (Sigma Aldrich Cat# C0549-1PT) extraction as recommended in the Biotin Labeling Kit protocol. Probe sequences are listed in Table 1.

**Table 1:** EMSA probe sequences.

Probe name	Sequence (5’ to 3’)
**High affinity (forward)**	CGGGCCAGCTGCTGTCTCGCTCGCACCCGCACCGCT
**High affinity (reverse)**	AGCGGTGCGGGTGCGAGCGAGACAGCAGCTGGCCCG
**Constant flank (forward)**	CTACTATAGCAATGGGAGCGAGAAGTATCAGTCAGT
**Constant flank (reverse)**	ACTGACTGATACTTCTCGCTCCCATTGCTATAGTAG
**Four-repeat (forward)**	GTATTGTTTATTTATGTAATTATAATGAGAGAGATATTGTTTATTTATTAATGTATAATT
**Four-repeat (reverse)**	AATTATACATTAATAAATAAACAATATCTCTCTCATTATAATTACATAAATAAACAATAC
**Eight-repeat (forward)**	TGTTTATTTATGTAATTATAATGAGAGAGAGAGAGAGATATTGTTTATTTATTAATGTAT
**Eight-repeat (reverse)**	ATACATTAATAAATAAACAATATCTCTCTCTCTCTCTCATTATAATTACATAAATAAACA
**Ten-repeat (forward)**	TTTATTTATGTAATTATAATGAGAGAGAGAGAGAGAGAGATATTGTTTATTTATTAATGT
**Ten-repeat (reverse)**	ACATTAATAAATAAACAATATCTCTCTCTCTCTCTCTCTCATTATAATTACATAAATAAA
**Fifteen-repeat (forward)**	TTATGTAATTATAATGAGAGAGAGAGAGAGAGAGAGAGAGAGAGATATTGTTTATTTATT
**Fifteen-repeat (reverse)**	AATAAATAAACAATATCTCTCTCTCTCTCTCTCTCTCTCTCTCTCATTATAATTACATAA

#### Electrophoretic mobility shift assay

EMSAs were performed using the Chemiluminescent Nucleic Acid Detection Module (Thermo Scientific Cat#89880)/LightShift Chemiluminescent EMSA Kit (Thermo Scientific Cat#20148). DNA binding conditions for CLAMP protein were optimized with 1 ug/uL Poly (dI•dC, Cat#20148E) and Elution Buffer: 5mM Tris-HCL, pH 8.5 (AE Macherey-Nagel Cat#740588.10). Control MBP protein was used from NEB and diluted to equal the molar concentration of CLAMP.

### ChIP-seq sample preparation and data analysis

#### Chromatin preparation

The following protocol is modified from Larschan et al., 2007. Chromatin was prepared from 150 sexed third instar larvae ground into a powder with a mortar and pestle while frozen in liquid nitrogen. Larvae were homogenized in a 40 mL Dounce homogenizer in 40 mLs of PBS containing a protease inhibitor cocktail (PI) (pancrease-extract 0.002 mg/ml, thermolysin 0.0005 mg/ml, chymotrypsin 0.002 mg/ml, trypsin 0.02 mg/ml, papain 0.33 mg/ml) (Roche). Next, the extract was crosslinked with 1% formaldehyde. Samples were rotated for 20 min at room temperature followed by quenching with 2 mLs 2.5 M glycine for 5 minutes at room temperature. The extract was chilled on ice and centrifuged for 5 minutes at 1500 g at 4°C. The pellet was resuspended in 40 mLs PBS containing 1x PI and 0.2 mM PMSF followed by centrifugation for 5 minutes at 1500 g at 4°C. Centrifugation and resuspension were repeated as described above with the following buffers: 1) 10 mLs Wash A (10 mM HEPES pH 7.6, 10 mM EDTA pH 8.0, 0.5 mM EGTA pH 8.0, 0.25% Triton-X 100, 1x PI, 0.2 mM PMSF); 2) 10 mLs Wash B (10 mM HEPES pH 7.6, 10 mM NaCl, 10 mM EDTA pH 8.0, 0.5 mM EGTA pH 8.0, 0.25% Triton-X 100, 1x PI, 0.2 mM PMSF); 3) 10 mLs TE Wash (10 mM Tris-HCL pH 8.0, 1 mM EDTA pH 8.0, 0.01% SDS, 1x PI, 0.2 mM PMSF); 4) 10 mLs TE Wash with SDS (10 mM Tris-HCl pH 8.0, 1 mM EDTA pH 8.0, 1% SDS, 1x PI, 0.2 mM PMSF), and 5) 2 washes with 10 mLs TE Wash. Next, the pellet was resuspended in 2 mLs Pre-RIPA (0.1% SDS, 10 mM Tris-HCL pH 8.0, Larval paste samples were sonicated on ice using a Bioruptor microtip sonicator for 6 30-second cycles at 20% amplitude, with one minute off in between cycles to prevent the sample from overheating. Samples were then cleaned up and run on a gel to validate sonication. To clean up the sonicated chromatin, 25 uL of sonicated sample was added to tubes containing 175 uL elution buffer (1% SDS, 0.1 M Sodium Bicarbonate), and crosslinks were reversed overnight at 65°C. 200 uL of TE buffer was added to each tube (0.01 M Tris, pH 9.0, 1 uM EDTA) along with 8 uL RNase A, and the mixture was incubated at 37°C for 2 hours. 4 uL Proteinase K was added per tube and samples were incubated at 55°C for two hours.

DNA was extracted using a Phenol-Chloroform-Isoamyl Alcohol (25:24:1) extraction using phase-lock tubes. The DNA was then precipitated with 5M NaCl, glycogen and ethanol at -80 for 1 hour and resuspended in 10 mL water. Precipitate was run on a 1% gel at 120 V for 1 hour and post-stained with Gel Red for 20 minutes. After validating the shearing, 100 uL Triton X-100, 20 uL 10% Sodium Deoxycholate, and 56 uL 5M NaCl were added. Samples were pelleted and the supernatant was saved. 50 uL was set aside as input.

#### Immunoprecipitation

For immunoprecipitation, 5 uL CLAMP antibody (Larschan et al 2012) was added to each sample and rotated at 4°C overnight. 100 uL Protein A Dynabeads per sample were washed in IP buffer (0.1% SDS, 10 mM Tris-HCl, pH 8.0, 1 mM EDTA, 1% Triton X-100, 0.1% Sodium Deoxycholate, 140 mM NaCl) and added to the IP. The lysate and beads were incubated for 2 hours at 4°C, then the beads were washed in wash buffer (50 mM HEPES-KOH, 0.1 M LiCl, 1 mM EDTA, 1% NP-40, 0.7% Sodium Deoxycholate). Samples were eluted in elution Buffer (1% SDS, 0.1 M Sodium Bicarbonate), and DNA was isolated as described above with a final resuspension into 50 μL water. DNA libraries were prepared using the Illumina TruSeq Kit, and sequenced on a Illumina HiSeq with 100-bp paired end sequencing.

#### ChIP-seq data analysis

Reads were aligned to the publically available assemblies for *D*. *melanogaster* (version dm3) and the *D*. *miranda* genome [15] allowing only unique alignments. The numbers of aligned reads from each sample are shown below. The positions with anomalously high tag counts were removed for the consideration as potential amplification artifacts [61]. Tag counts were normalized by the corresponding library sizes and enrichments were calculated as ratios of normalized tag counts in ChIP and input samples. Peaks in the ChIP-Seq tag distribution were called using the SPP software package with default parameters. Overlapping peaks of two replicates were used in the analysis. Data are available with the following NCBI GEO accession number: GSE83435.

#### Alignment results

*D*. *melanogaster* replicate 1 input: 25208995 reads were processed, 17035648 (67.58%) were aligned.

*D*. *melanogaster* replicate 1 ChIP: 10298719 reads were processed, 5882185 (57.12%) were aligned.

*D*. *melanogaster* replicate 2 input: 16543983 reads were processed, 12299524 (74.34%) were aligned.

*D*. *melanogaster* replicate 2 ChIP: 5639720 reads were processed, 267213 (47.38%) were aligned.

*D*. *miranda* replicate 1 input: 9544768 reads were processed, 3234724 (33.89%) were aligned.

*D*. *miranda* replicate 1 ChIP: 8751503 reads were processed, 3149806 (35.99%) were aligned.

*D*. *miranda* replicate 2 input: 4782344 reads were processed, 2123634 (44.41%) were aligned.

*D*. *miranda* replicate 2 ChIP: 15175635 reads were processed, 5512100 (36.32%) were aligned.

### Analysis of CLAMP peak clustering

CLAMP binding sites (ChIP-seq peaks from Soruco *et al*. 2013) were considered in this analysis. We measured the distance of each binding site to its neighbors and calculated average distance up to nth neighbor. We did the same calculation for randomized peaks and compared distance of CLAMP binding sites to the median of 10 randomized sets. We considered binding sites are around CES if they are within the median distance value of chromosome X.

### Motif representation and comparison

Nucleotide frequency in the motif is shown in log scale as ‘bits’; the maximum value is log_2_ 4 = 2 bits, where 4 is the number of nucletotide types. The significance of the motif is represented with an *E*-value score calculated by MEME. The *E*-value is an estimate of the expected number of motifs with the given log likelihood ratio (or higher), and with the same width and site count, that one would find in a similarly sized set of random sequences.

To compare motifs, we calculated the Euclidean distance of frequencies which is defined as the-root-mean-square-difference of nucleotide frequencies for each position.

### Analysis of motif density within genomes

To determine CLAMP binding site motif density on chromosomes, sequences that match the motif were identified on chromosomes and exact matches were counted as hits. For repeat density analysis, repeats with different lengths were identified on chromosomes and the number of hits were normalized by the length of each chromosome.

## Supporting Information

S1 Fig **A)** 'PBM+ChIP+MRE+', 'PBM+ChIP+MRE-', 'PBM-ChIP-MRE+', 'PBM-ChIP-MRE-', 'PBM-ChIP+MRE+', 'PBM-ChIP+MRE-', 'PBM+ChIP-MRE+' and ‘PBM+ChIP-MRE-' groups are given with their *E*-values and number of sites. **B)** CLAMP ChIP-seq enrichment is shown for the sequences similar to the custom PBM CLAMP motif found. Similarity of the *in vivo* sequences to the PBM motif was calculated using the FIMO tool.
(PDF)

S2 Fig **A)** Quantiles of 'PBM+ChIP+MRE+', 'PBM+ChIP+MRE-', 'PBM-ChIP-MRE+' and 'PBM-ChIP-MRE-' groups are given. Groups are divided into five different quantiles based on binding intensity scores and motifs were obtained from the sequences in each quantile. **B)** Euclidean distances between quantiles are shown for 8-bp core and flanking parts separately. Average Euclidean distances were calculated for each part of the motif.
(PDF)

S3 Fig Kolmogorov–Smirnov test (KS-test) between autosomes and chromosome X binding site DNA shape profiles in the strong (A) and the weak (B) groups. Black asterisks indicate significant different with p-value < 0.05, and red asterisks with p-value < 0.001.
(PDF)

S4 Fig CLAMP ChIP-seq enrichment on GA repeats of different lengths is shown for S2 (A) and Kc (B) 2L, 2R, 3L and 3R autosomal arms. (PDF)

S5 Fig **A)** PBM intensities of probes selected from autosomes and the X-chromosome are plotted for each quantile. *p*-values calculated via Kolmogorov–Smirnov test are given for each pair. **B)** Density of dinucleotide repeats on the *D*. *melanogaster* chromosome X is compared with chromosomes 2L, 2R, 3L and 3R individually.
(PDF)

S6 Fig **A)** Average distance between neighboring S2 CLAMP ChIPseq peaks is plotted (top panel). Values are normalized based on a random distribution of peaks (bottom panel). **B)** The same average plots shown for S2 cells in (A) are shown for Kc cells.
(PDF)

S7 Fig **A)** Ratio of CLAMP motif hit density in the gene body (TSS+250bp-TTS) to 5’ end (TSS+/-250-bp) is shown for each chromosomal arm; values are normalized to the X-chromosome value. **B)** The same analysis conducted for part A is shown for S2 and Kc CLAMP ChIP-seq peaks instead of motifs.
(PDF)

S8 Fig A schematic of the CLAMP (CG1832) gene from *D*. *melanogaster* is shown in blue. Conservation of the CLAMP gene sequence compared to orthologues in other Drosophilids and the mosquito (*A*. *gambiae*) is shown below (UCSC Genome Browser).
(PDF)

S9 Fig Amino acid multiple alignment of CLAMP from *A*. *gambiae*, *D*. *miranda*, and *D*. *melanogaster* using ClustalOmega [34]. Colors and symbols indicate residue properties and conservation: Red = small and hydrophilic, Blue = acidic, Magenta = basic, Green = Hydroxyl, sulfhydryl, amine, or glycine, * = single, fully conserved residue,: = strongly similar residues,. = weakly similar residues.
(PDF)

S10 Fig Density ratio of neoX (Muller-C), XL (Muller-A), and XR (Muller-D) chromosomes to autosomes in *D*. *miranda* and X (Muller-A) chromosome to autosome in *A*. *gambiae* are shown for different types of dinucleotide repeats.
(PDF)

S11 Fig Density ratio of XL (Muller-A), XR (Muller-D) and neoX (Muller-C) chromosomes to chromosomes 2 (Muller-B), 4 (Muller-E) and 5 (Muller-F) in *D*. *miranda* are shown for different repeats. (PDF)

S12 Fig Density ratio of X (Muller-A) chromosome to chromosomes 2L (Muller-D), 2R (Muller-E), 3L (Muller-C) and 3R (Muller-B) in *A*. *gambiae* are shown for different repeats. (PDF)

S13 Fig **A)** CLAMP ChIP-seq hit density per 1Mb is shown for each chromosome of *D*. *miranda*. Similarity of the genomic sequence under each peak to the ChIP-seq motif (Fig 6C) was calculated using the FIMO tool. **B)** The motif from the top 1000 ChIP-seq peaks (top panel) was compared with the custom PBM motif. Sequences that include the custom PBM motif are presented as the custom PBM motif hits (middle panel). Euclidean distances between the top and middle motifs are given for each position within the PWM (bottom panel). **C)** As the similarity to an *in vivo* motif increases (lower p-value), the occurrence of the motifs increases in called peaks vs. randomized and scrambled peaks for both *D*.*melanogaster* and *D*. *miranda*. **D)** Motif occurrence presented in part C is shown as the ratio of X-chromosome to autosomes.
(PDF)

S14 Fig Amino acid pairwise alignments using EMBOSS Needle [38] of the *D*. *melanogaster* and *D*. *miranda* MSL2 CXC domain. Symbols indicated conserved residues: | = fully conserved,: = similar,. = mismatch.
(PDF)

S1 Table The PBM probes. A description of the PBM probe classes and the number of probes in each class.
(PDF)

S2 Table Kolmogorov–Smirnov test was applied to the PBM intensities of probes. Categories tested include ‘ChIP+MRE+’, ‘ChIP+MRE-’, ‘ChIP-MRE+’, ‘ChIP-MRE-’, ‘PBM+ChIP+MRE+’, ‘PBM+ChIP+MRE-’, ‘PBM-ChIP-MRE+’, and ‘PBM-ChIP-MRE-’, where ‘ChIP+’ indicates CLAMP binding *in vivo*, ‘MRE+’ indicates the sequence is with MSL recognition element and ‘PBM+’ indicates CLAMP binding *in vitro*. Values show the p-values.
(PDF)

S3 Table Kolmogorov–Smirnov test was applied to the enrichments on the sequences with different similarities to the motif. Values show the Kolmogorov–Smirnov test p-values. Similarities to the motif were calculated with the FIMO tool, and categories are–log10(p-value) is smaller than 4, between 4 and 5, between 5 and 6, and larger than 6.
(PDF)

S4 Table Euclidean distance between motifs. The Euclidean distance between all classes of motifs is shown. The Euclidean distance between the 8-bp cores and the flanking sequences are shown separately.
(PDF)

S5 Table Kolmogorov-Smirnov test to compare binding to different flanking sequence categories. Kolmogorov–Smirnov test was applied to the PBM intensities of probes with 8-bp core part and matched endogenous flank, with 8-bp core part and unmatched endogenous flank, with 8-bp core part and unmatched synthetic flank, without 8-bp core part, and with 8-bp core part and matched endogenous flank for 4 zinc finger protein. Values show the p-values.
(PDF)

S6 Table The count of probes that contain each number of GA-repeats. (PDF)

S7 Table Number of GA-repeats in *D*. *melanogaster* autosomes (A) and the X-chromosome (X). Repeats are not overlapping, i.e. the repeats are assigned by the longest length. For the two replicates used in Fig 3D, these numbers are doubled.
(PDF)

S8 Table Number of GA repeats in *D*. *melanogaster* chromosomes and individual chromosomal arms. Repeats with different lengths may be overlapping.
(PDF)

S9 Table Kolmogorov–Smirnov test was applied to the average distance values between S2 CLAMP ChIP-seq peaks. (PDF)

S10 Table Kolmogorov–Smirnov test was applied to the average distance values between Kc CLAMP ChIP-seq peaks. (PDF)

S11 Table Numbers of S2 and KC ChIP-seq peaks used in the average distance calculation are given. (PDF)

S12 Table Kolmogorov–Smirnov test was applied to the normalized distance values between S2 CLAMP ChIP-seq peaks. (PDF)

S13 Table Kolmogorov–Smirnov test was applied to the normalized distance values between Kc CLAMP ChIP-seq peaks. (PDF)

S14 Table Conservation of CLAMP across species. Conservation of CLAMP relative to *D*. *melanogaster* was determined for both the full-length protein (FL) and the DNA binding domain (DBD) alone.
(PDF)

S15 Table Number of matches to the CLAMP PBM motif per Mb on individual chromosomal arms. (PDF)

S16 Table Number of GA repeats in *D*. *miranda* chromosomes. Repeats may be overlapping.
(PDF)

S17 Table Number of GA repeats in *A*. *gambiae* chromosomes. Repeats may be overlapping.
(PDF)
