# Supplementary figures and images for: Expansion of GA Dinucleotide Repeats Increases the Density of CLAMP Binding Sites on the X-Chromosome to Promote Drosophila Dosage Compensation
Source: PLoS Genet. 2016 Jul 14;12(7):e1006120. doi: 10.1371/journal.pgen.1006120 (PMC4945028; doi:10.1371/journal.pgen.1006120)

**A**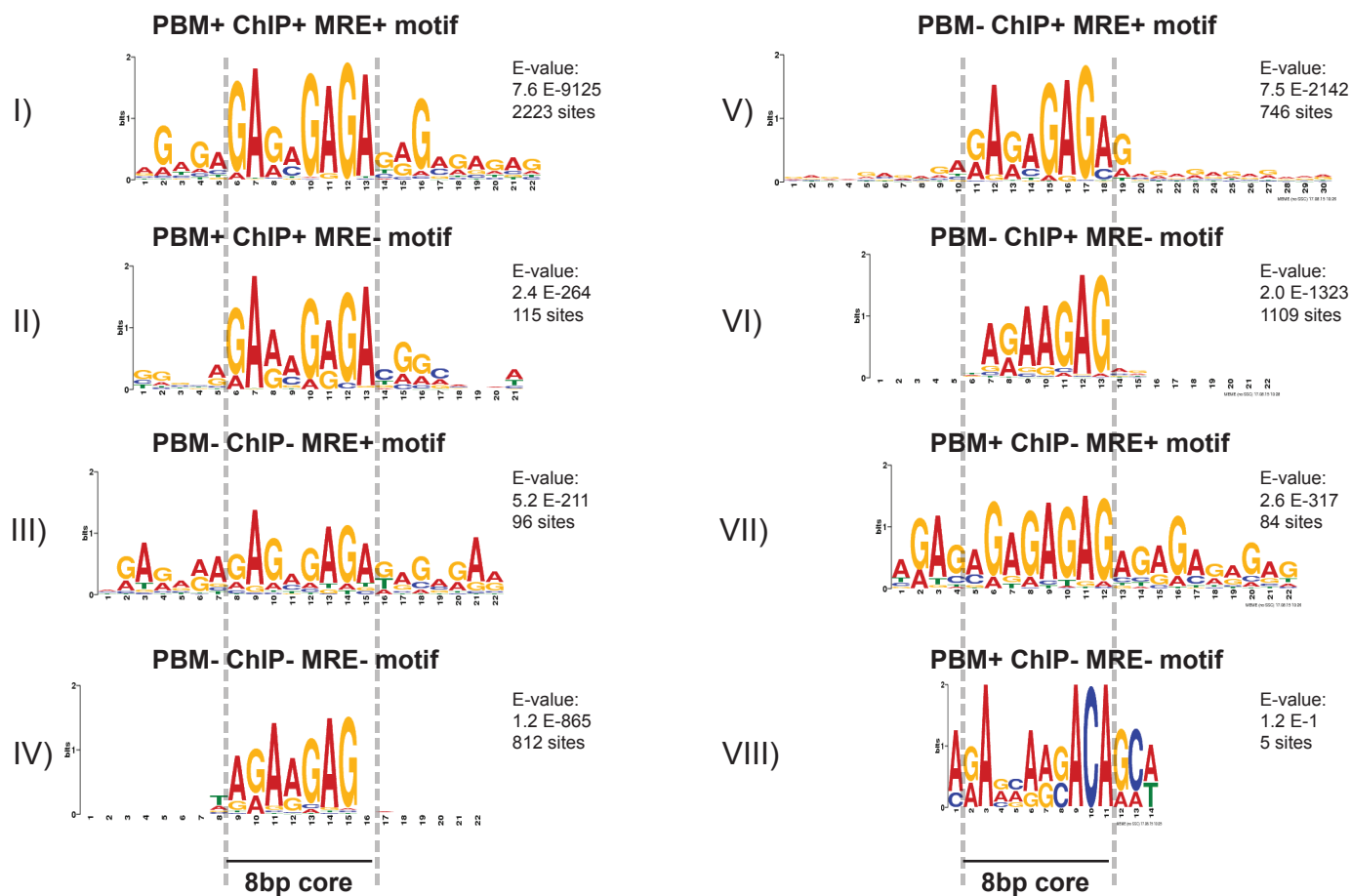**B**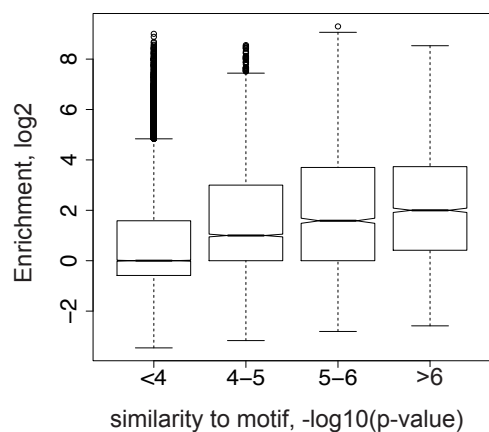

Supplement: S1 Fig — A) 'PBM+ChIP+MRE+', 'PBM+ChIP+MRE-', 'PBM-ChIP-MRE+', 'PBM-ChIP-MRE-', 'PBM-ChIP+MRE+', 'PBM-ChIP+MRE-', 'PBM+ChIP-MRE+' and ‘PBM+ChIP-MRE-' groups are given with their E-values and number of sites. B) CLAMP ChIP-seq enrichment is shown for the sequences similar to the custom PBM CLAMP motif found. Similarity of the in vivo sequences to the PBM motif was calculated using the FIMO tool. (PDF) [file pgen.1006120.s001.pdf]

**A** Quantiles of the motif sets

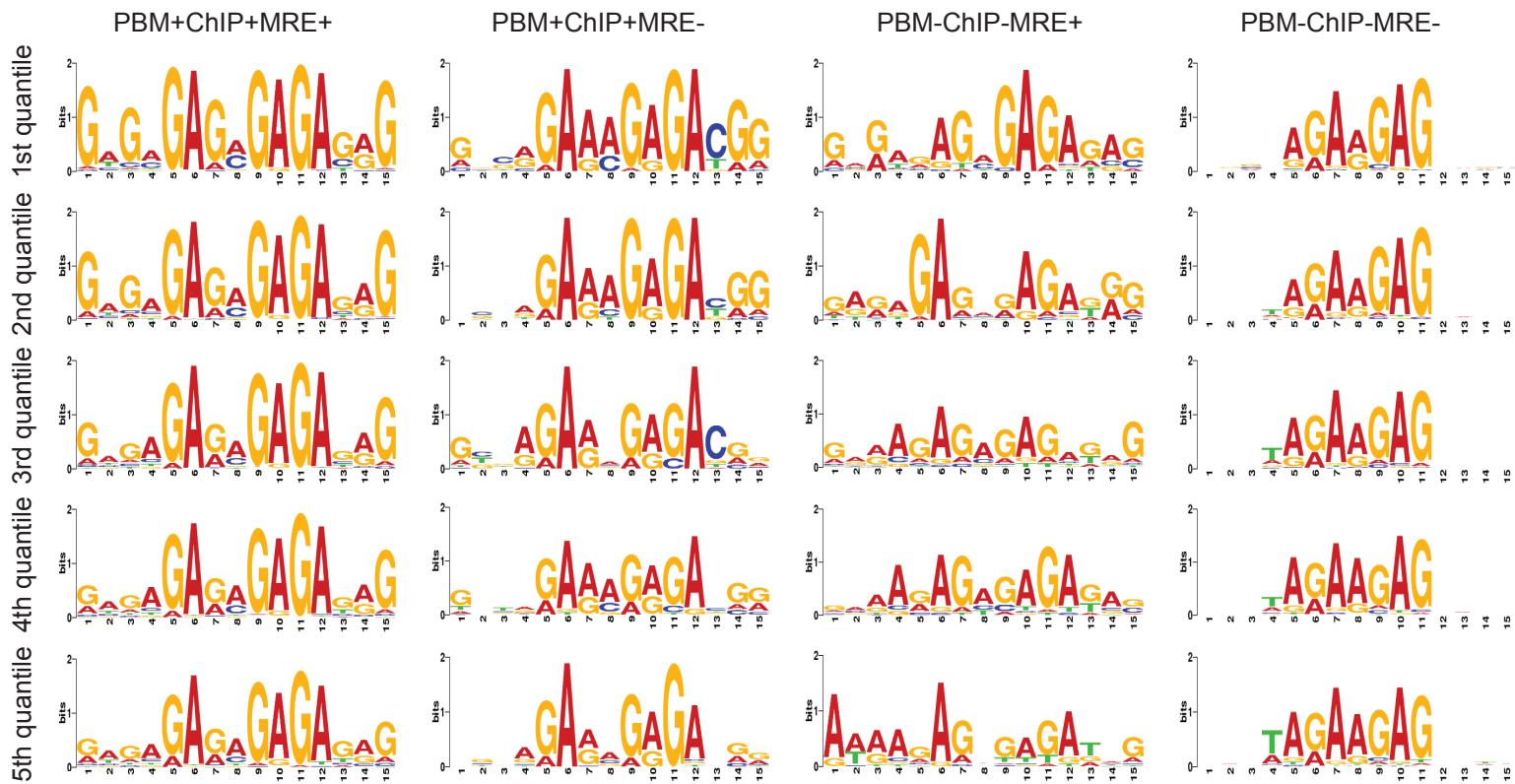

**B** Euclidean Distance among quantiles

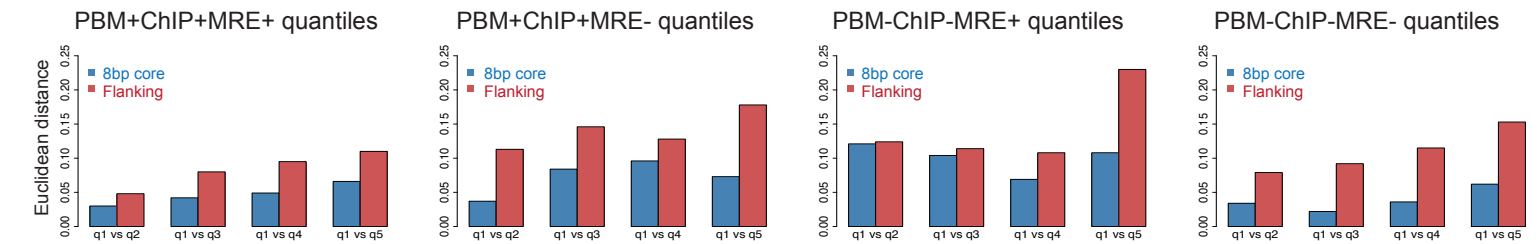

Supplement: S2 Fig — A) Quantiles of 'PBM+ChIP+MRE+', 'PBM+ChIP+MRE-', 'PBM-ChIP-MRE+' and 'PBM-ChIP-MRE-' groups are given. Groups are divided into five different quantiles based on binding intensity scores and motifs were obtained from the sequences in each quantile. B) Euclidean distances between quantiles are shown for 8-bp core and flanking parts separately. Average Euclidean distances were calculated for each part of the motif. (PDF) [file pgen.1006120.s002.pdf]

**A**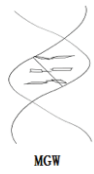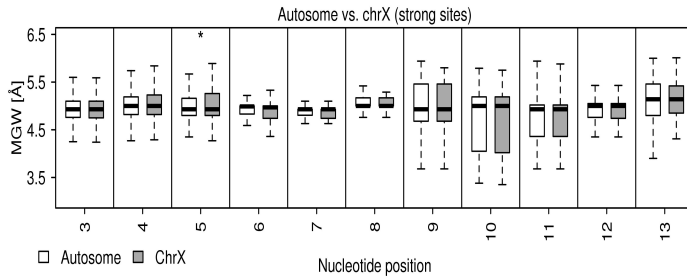**B**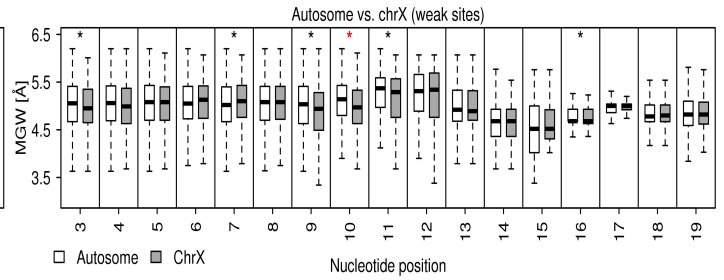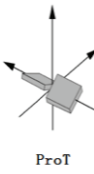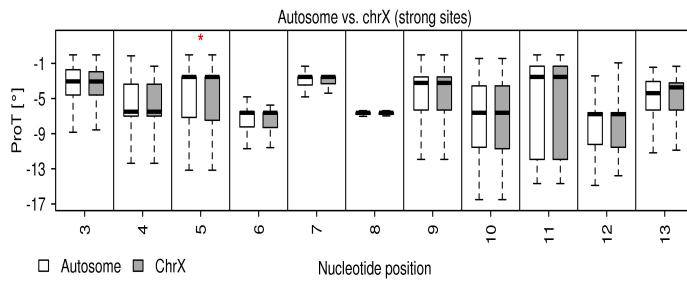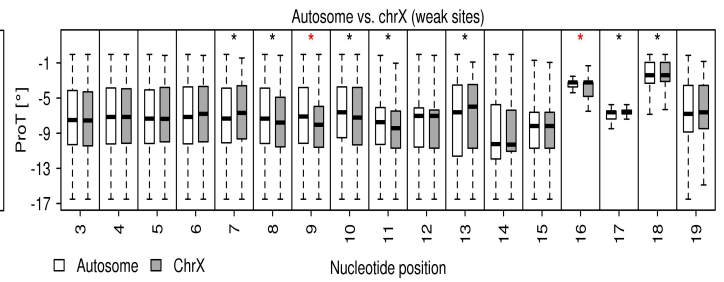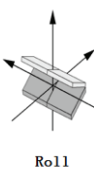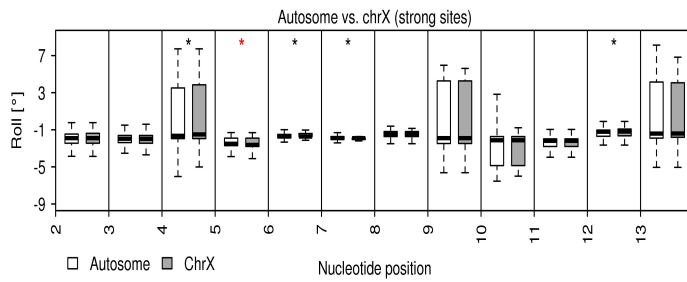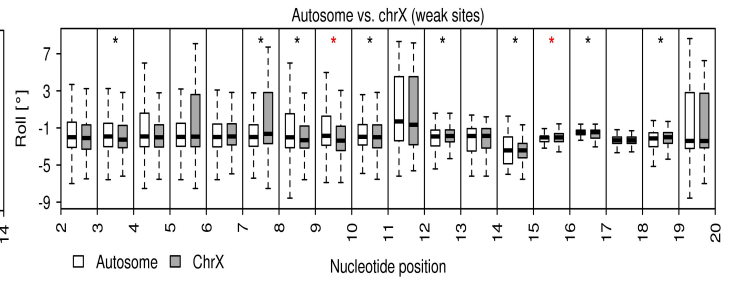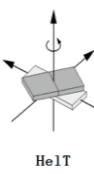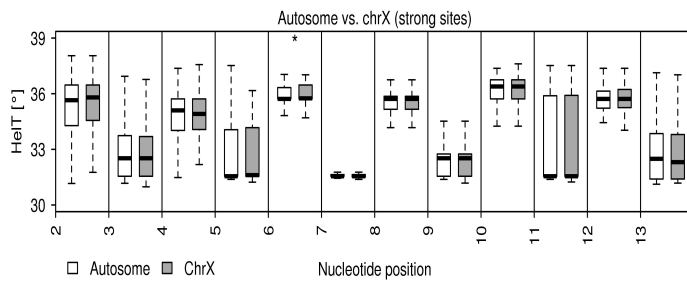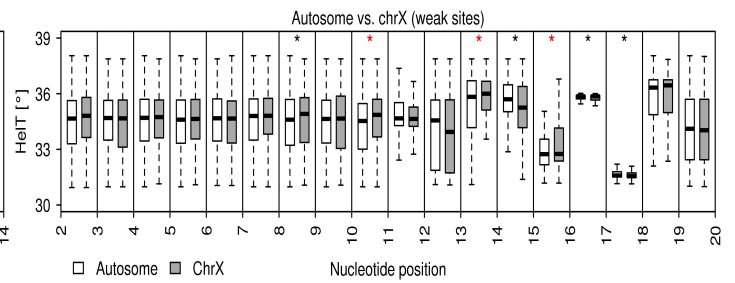

Supplement: S3 Fig — Black asterisks indicate significant different with p-value < 0.05, and red asterisks with p-value < 0.001. (PDF) [file pgen.1006120.s003.pdf]

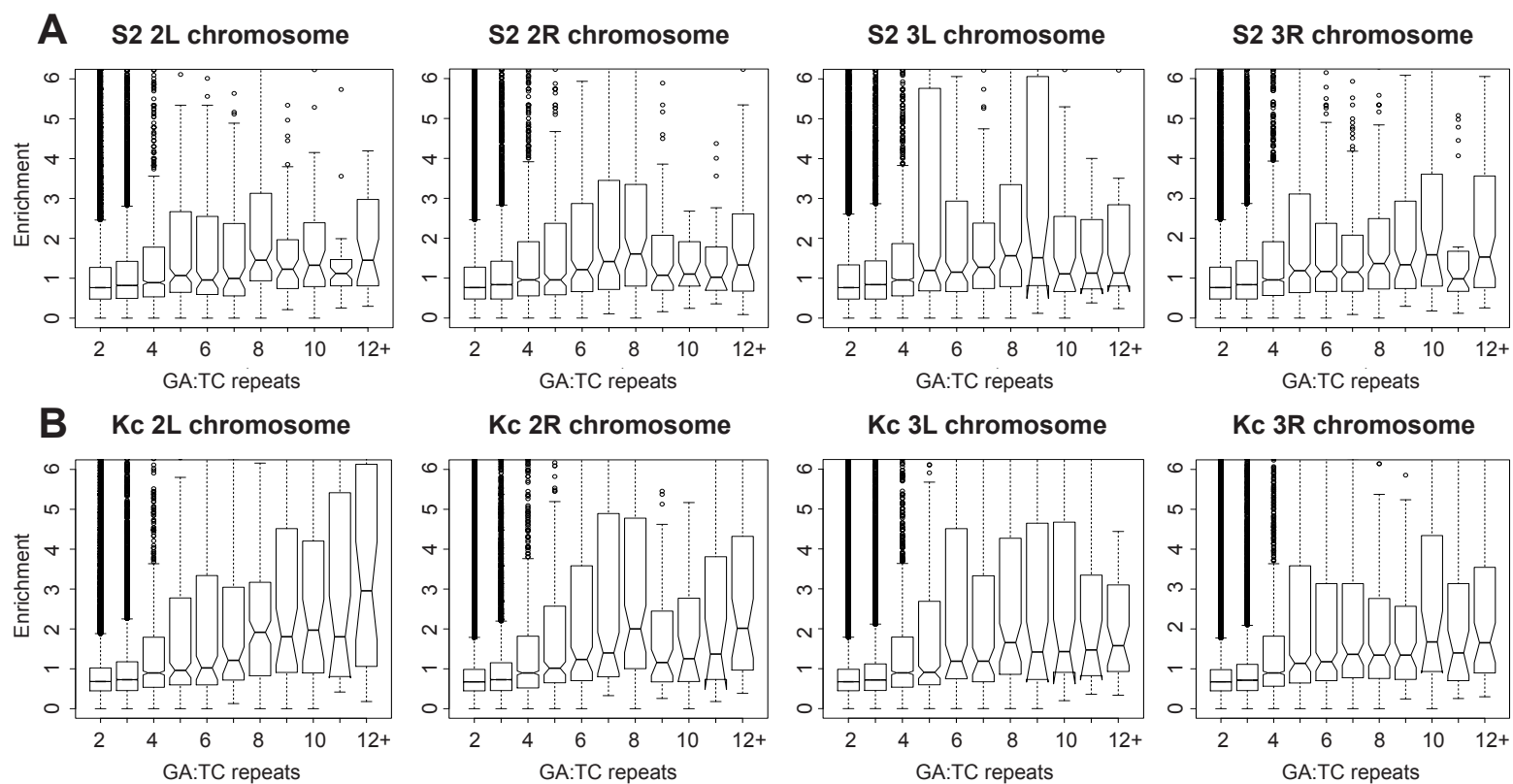

Supplement: S4 Fig — (PDF) [file pgen.1006120.s004.pdf]

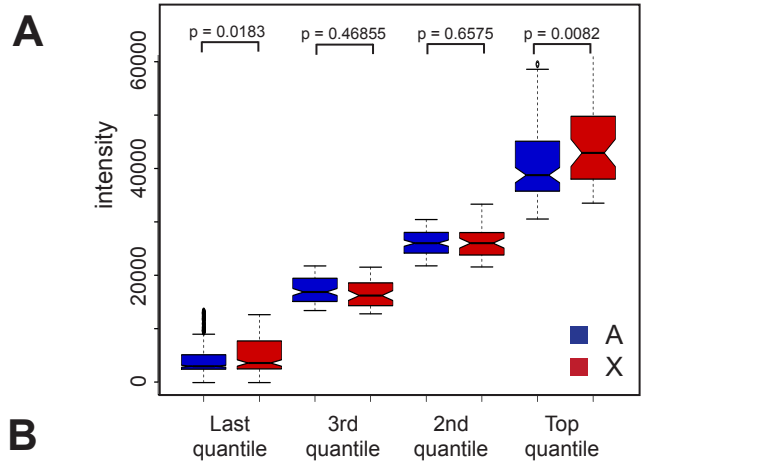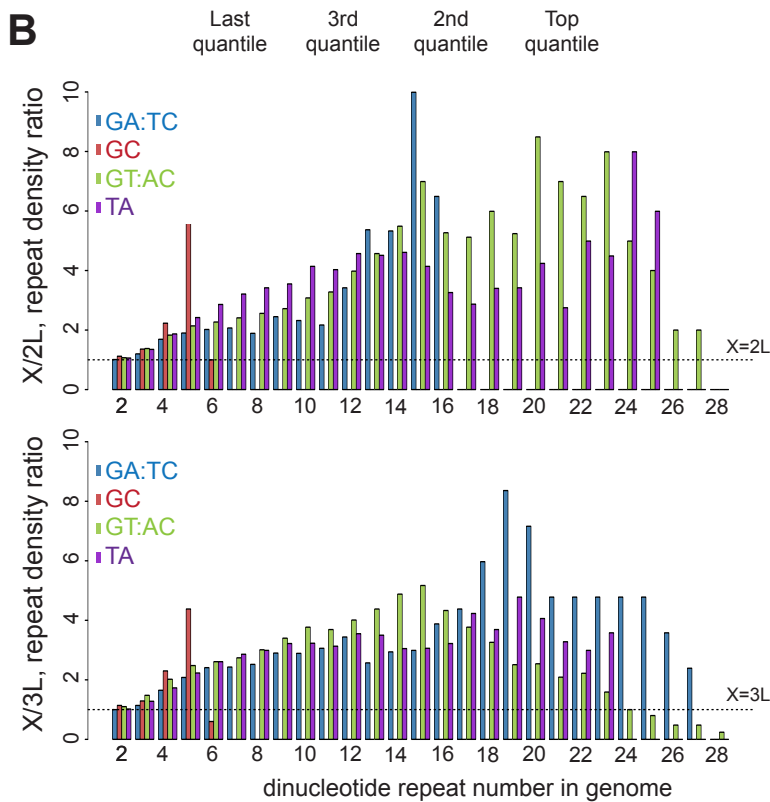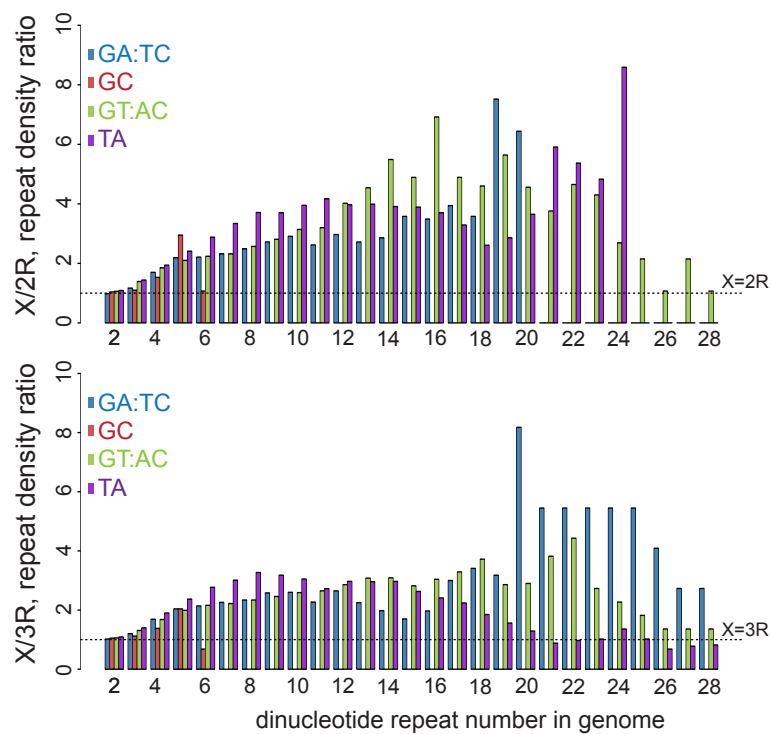

Supplement: S5 Fig — A) PBM intensities of probes selected from autosomes and the X-chromosome are plotted for each quantile. p-values calculated via Kolmogorov–Smirnov test are given for each pair. B) Density of dinucleotide repeats on the D. melanogaster chromosome X is compared with chromosomes 2L, 2R, 3L and 3R individually. (PDF) [file pgen.1006120.s005.pdf]

**A**

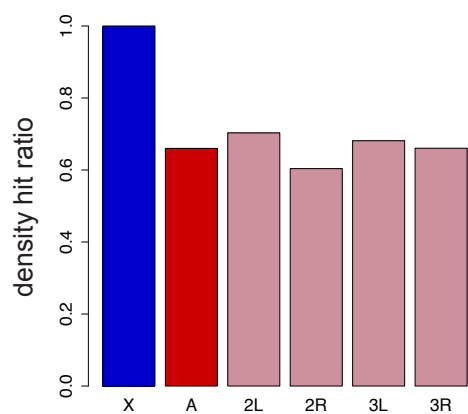

**B**

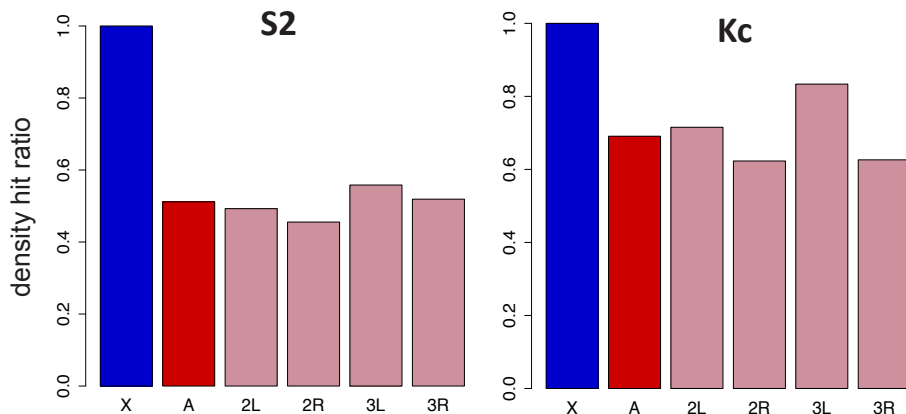

Supplement: S7 Fig — A) Ratio of CLAMP motif hit density in the gene body (TSS+250bp-TTS) to 5’ end (TSS+/-250-bp) is shown for each chromosomal arm; values are normalized to the X-chromosome value. B) The same analysis conducted for part A is shown for S2 and Kc CLAMP ChIP-seq peaks instead of motifs. (PDF) [file pgen.1006120.s007.pdf]

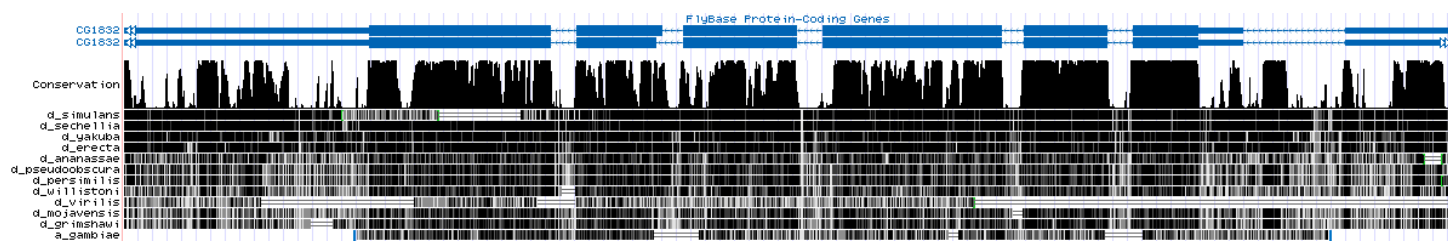

Supplement: S8 Fig — Conservation of the CLAMP gene sequence compared to orthologues in other Drosophilids and the mosquito (A. gambiae) is shown below (UCSC Genome Browser). (PDF) [file pgen.1006120.s008.pdf]

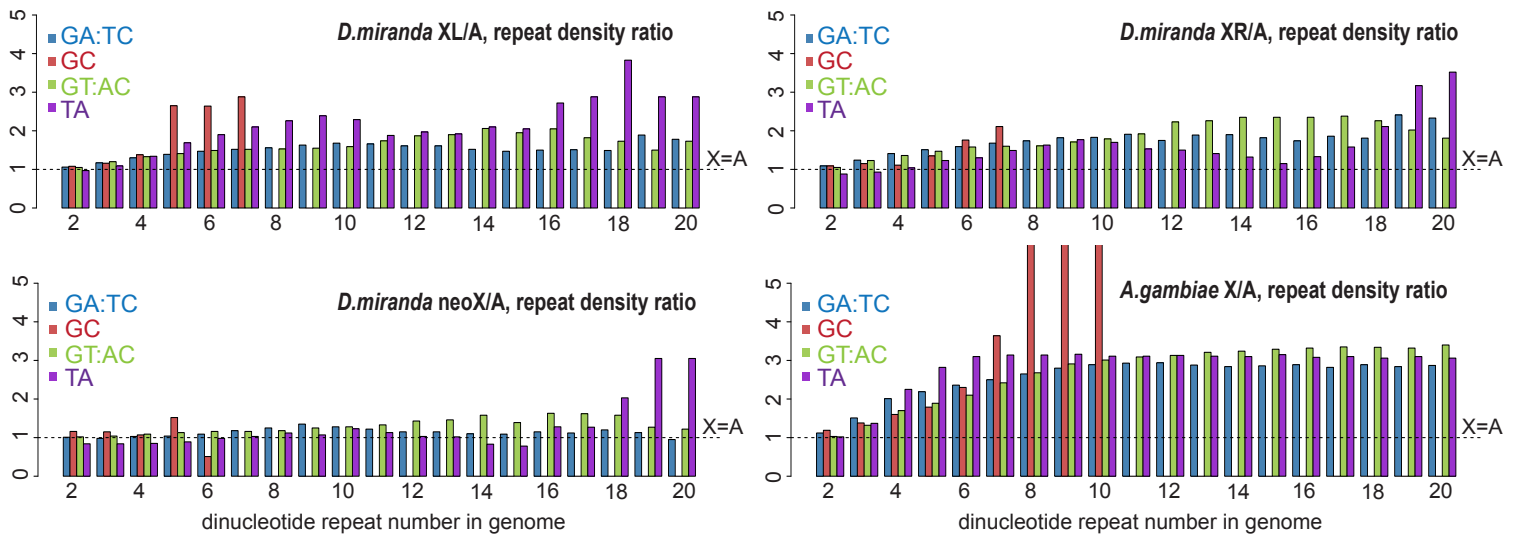

Supplement: S10 Fig — gambiae are shown for different types of dinucleotide repeats. (PDF) [file pgen.1006120.s010.pdf]

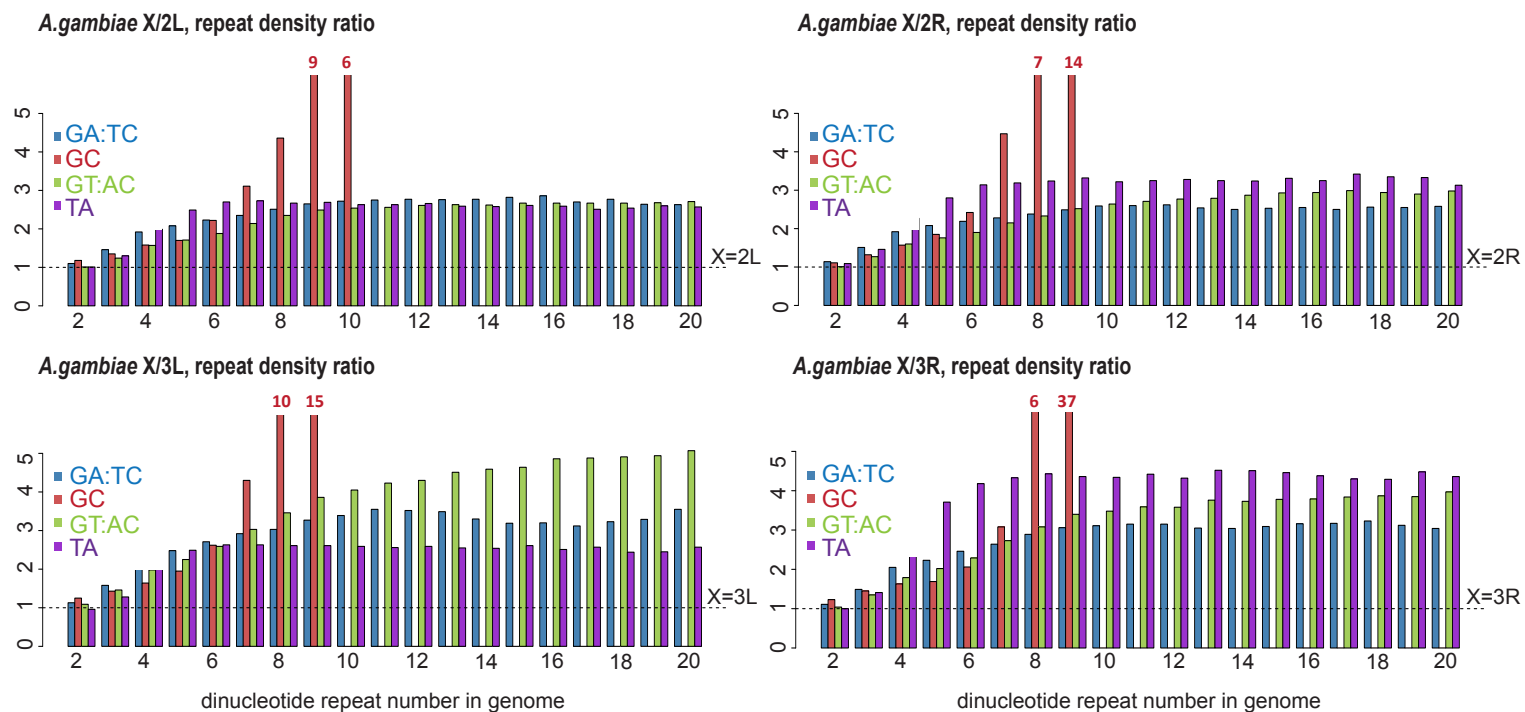

Supplement: S12 Fig — (PDF) [file pgen.1006120.s012.pdf]

**A**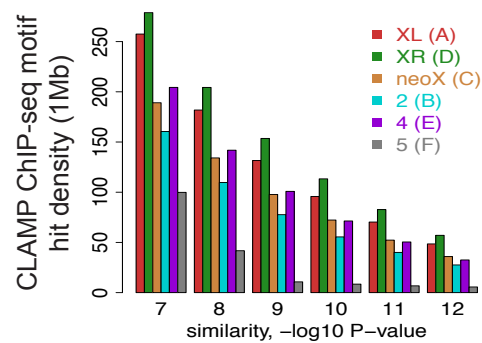**B**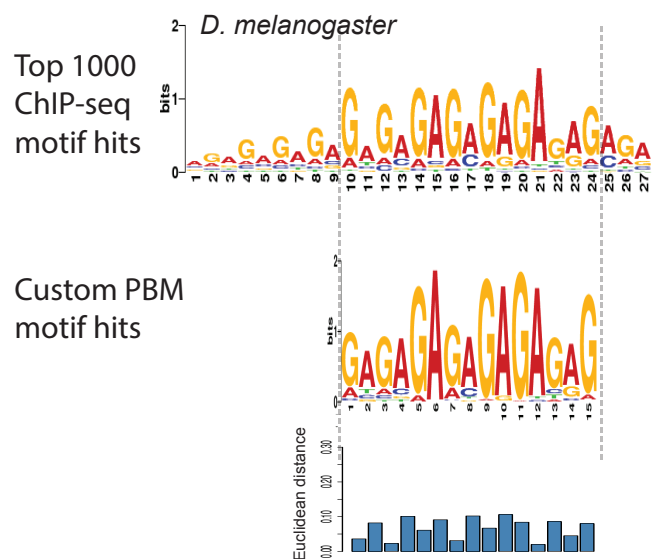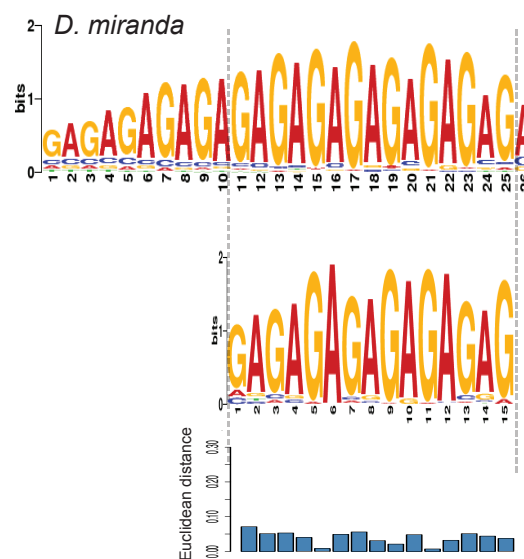**C****Occurrence of motifs**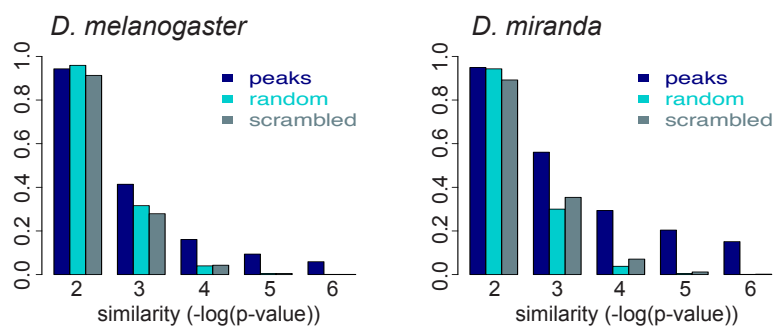**D****Occurrence of motifs, X/A**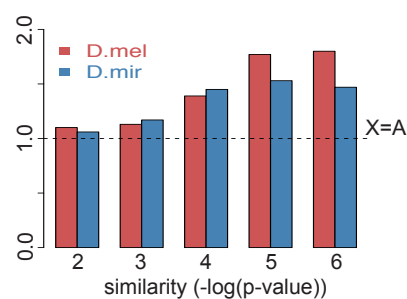

Supplement: S13 Fig — A) CLAMP ChIP-seq hit density per 1Mb is shown for each chromosome of D. miranda. Similarity of the genomic sequence under each peak to the ChIP-seq motif (Fig 6C) was calculated using the FIMO tool. B) The motif from the top 1000 ChIP-seq peaks (top panel) was compared with the custom PBM motif. Sequences that include the custom PBM motif are presented as the custom PBM motif hits (middle panel). Euclidean distances between the top and middle motifs are given for each position within the PWM (bottom panel). C) As the similarity to an in vivo motif increases (lower p-value), the occurrence of the motifs increases in called peaks vs. randomized and scrambled peaks for both D.melanogaster and D. miranda. D) Motif occurrence presented in part C is shown as the ratio of X-chromosome to autosomes. (PDF) [file pgen.1006120.s013.pdf]
