# Supplementary material for: Expansion of GA Dinucleotide Repeats Increases the Density of CLAMP Binding Sites on the X-Chromosome to Promote Drosophila Dosage Compensation
Source: PLoS Genet. 2016 Jul 14;12(7):e1006120. doi: 10.1371/journal.pgen.1006120 (PMC4945028; doi:10.1371/journal.pgen.1006120)

Figure S6 (Kuzu, Kaye et al.)

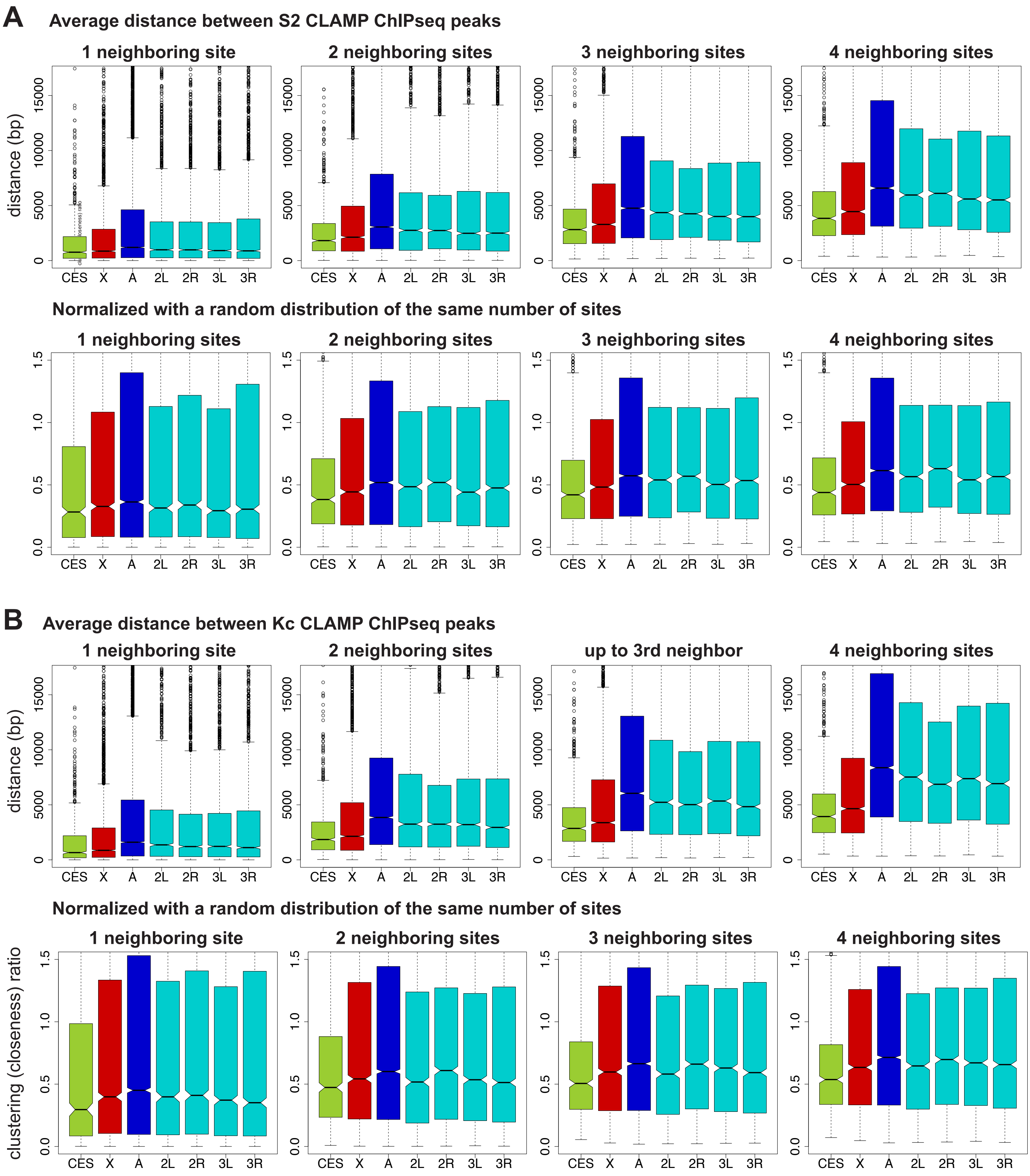

Supplement: S6 Fig — A) Average distance between neighboring S2 CLAMP ChIPseq peaks is plotted (top panel). Values are normalized based on a random distribution of peaks (bottom panel). B) The same average plots shown for S2 cells in (A) are shown for Kc cells. (PDF) [file pgen.1006120.s006.pdf]
