# Supplementary material for: Expansion of GA Dinucleotide Repeats Increases the Density of CLAMP Binding Sites on the X-Chromosome to Promote Drosophila Dosage Compensation
Source: PLoS Genet. 2016 Jul 14;12(7):e1006120. doi: 10.1371/journal.pgen.1006120 (PMC4945028; doi:10.1371/journal.pgen.1006120)
Supplement: S14 Fig — Symbols indicated conserved residues: | = fully conserved,: = similar,. = mismatch. (PDF) [file pgen.1006120.s014.pdf]

```
Mel_MSL2_CXC  525 CRCGISGSSNTLTTCRNSRCPCYKSYNSCAGCHCVCC 583
                             |....|     ||||.|:||.|.|..|
Mir_MSL2_CXC  364 -----------TPAARR-----CYKSGNTCANCRCFGC 385
```
