# Supplementary material for: Expansion of GA Dinucleotide Repeats Increases the Density of CLAMP Binding Sites on the X-Chromosome to Promote Drosophila Dosage Compensation
Source: PLoS Genet. 2016 Jul 14;12(7):e1006120. doi: 10.1371/journal.pgen.1006120 (PMC4945028; doi:10.1371/journal.pgen.1006120)
Supplement: S1 Table — A description of the PBM probe classes and the number of probes in each class. (PDF) [file pgen.1006120.s015.pdf]

Table S1. The PBM probes.

Probe ID	# of Probes	# Unique Sequences	# of Replicates	Brief Desc.
**(A) PROBES EXAMINING SINGLE pMRE/MRE SITES**				
mreP_gen_nX_oY_rZ	27216	3402	4	ChIP, +MRE/+pMRE, Genomic flanks
mreN_gen_nX_oY_rZ	10168	1271	4	ChIP, -MRE/+pMRE, Genomic flanks
negP_gen_nX_oX_rX	1664	208	4	(Neg Control) No ChIP, +MRE/+pMRE, Genomic flanks
negN_gen_nX_oX_rX	6576	822	4	(Neg Control) No ChIP, -MRE/+pMRE, Genomic flanks
cons_nX_oY_rZ	2816	352	4	Aggregate probeset of the 8-11bp pMREs with Constant Flanks
**(B) PROBES TILING ACROSS DIFFERENT GENOMIC REGIONS**				
rox1_n0_pX_oX_rX	1648	206	4	Wide Tiling (1000bp windows), High Affinity Region
5c2_n0_pX_oX_rX	1632	204	4	Wide Tiling (1000bp windows), High Affinity Region
rox2_n0_pX_oX_rX	1648	206	4	Wide Tiling (1000bp windows), High Affinity Region
11d1_n0_pX_oX_rX	1648	206	4	Wide Tiling (1000bp windows), High Affinity Region
15a8_n0_pX_oX_rX	1648	206	4	Wide Tiling (1000bp windows), High Affinity Region
til_nochp_nX_pX_oX_rX	16456	2057	4	Wide Tiling (1000bp windows), No CLAMP Chip, +pMRE
til_chpmre_nX_pX_oX_rX	19568	2446	4	Wide Tiling (1000bp windows) CLAMP Chip, +MRE/pMRE
til_chpno_nX_pX_oX_rX	16424	2053	4	Wide Tiling (1000bp windows) CLAMP Chip, -MRE/-pMRE
ntil_nochp_nX_pX_oX_rX	14104	1763	4	Narrow Tiling (100bp windows), CLAMP Chip, -MRE/-pMRE
ntil_chp_nX_pX_oX_rX	33720	4215	4	Narrow Tiling (100bp windows), CLAMP Chip, +MRE/+pMRE
**(C) CONTROL PROBES**				
pX_pX_nX_oX_rX	540	90	6	Pairs of Sites
mre1_PO_pX_wX_oX_rX	336	28	6	Position Dependence (PWM1:AGAGAGAGA)
mre2_PO_pX_wX_oX_rX	348	29	6	Position Dependence (PWM2:TAGAAGAG)
extra_nX_oX_rX	570	6	48	
