# Supplementary material for: Expansion of GA Dinucleotide Repeats Increases the Density of CLAMP Binding Sites on the X-Chromosome to Promote Drosophila Dosage Compensation
Source: PLoS Genet. 2016 Jul 14;12(7):e1006120. doi: 10.1371/journal.pgen.1006120 (PMC4945028; doi:10.1371/journal.pgen.1006120)
Supplement: S2 Table — Categories tested include ‘ChIP+MRE+’, ‘ChIP+MRE-’, ‘ChIP-MRE+’, ‘ChIP-MRE-’, ‘PBM+ChIP+MRE+’, ‘PBM+ChIP+MRE-’, ‘PBM-ChIP-MRE+’, and ‘PBM-ChIP-MRE-’, where ‘ChIP+’ indicates CLAMP binding in vivo, ‘MRE+’ indicates the sequence is with MSL recognition element and ‘PBM+’ indicates CLAMP binding in vitro. Values show the p-values. (PDF) [file pgen.1006120.s016.pdf]

**Table S2.** Kolmogorov–Smirnov test was applied to the PBM intensities of probes.

| | ChIP+ MRE+ | ChIP+ MRE- | ChIP- MRE+ | ChIP- MRE- | PBM+ ChIP+ MRE+ | PBM+ ChIP+ MRE- | PBM+ ChIP+ MRE+ | PBM+ ChIP+ MRE- |
|---|---|---|---|---|---|---|---|---|
| ChIP+ MRE+ | | | | | | | | |
| ChIP+ MRE- | < 2.2e-16 | | | | | | | |
| ChIP- MRE+ | 2.51e-13 | < 2.2e-16 | | | | | | |
| ChIP- MRE- | < 2.2e-16 | < 2.2e-16 | < 2.2e-16 | | | | | |
| PBM+ ChIP+ MRE+ | < 2.2e-16 | < 2.2e-16 | < 2.2e-16 | < 2.2e-16 | | | | |
| PBM+ ChIP+ MRE- | 1.29e-06 | < 2.2e-16 | 8.31e-10 | < 2.2e-16 | 1.92e-13 | | | |
| PBM- ChIP- MRE+ | < 2.2e-16 | 2.87e-12 | 1.73e-10 | < 2.2e-16 | < 2.2e-16 | < 2.2e-16 | | |
| PBM- ChIP- MRE- | < 2.2e-16 | < 2.2e-16 | < 2.2e-16 | 1 | < 2.2e-16 | < 2.2e-16 | < 2.2e-16 | |

Categories tested include ‘ChIP+MRE+’, ‘ChIP+MRE-’, ‘ChIP-MRE+’, ‘ChIP-MRE-’, ‘PBM+ChIP+MRE+’, ‘PBM+ChIP+MRE-’, ‘PBM-ChIP-MRE+’, and ‘PBM-ChIP-MRE-’, where ‘ChIP+’ indicates CLAMP binding *in vivo*, ‘MRE+’ indicates the sequence is with MSL recognition element and ‘PBM+’ indicates CLAMP binding *in vitro*. Values show the p-values.
