# Supplementary material for: Expansion of GA Dinucleotide Repeats Increases the Density of CLAMP Binding Sites on the X-Chromosome to Promote Drosophila Dosage Compensation
Source: PLoS Genet. 2016 Jul 14;12(7):e1006120. doi: 10.1371/journal.pgen.1006120 (PMC4945028; doi:10.1371/journal.pgen.1006120)
Supplement: S3 Table — Values show the Kolmogorov–Smirnov test p-values. Similarities to the motif were calculated with the FIMO tool, and categories are–log10(p-value) is smaller than 4, between 4 and 5, between 5 and 6, and larger than 6. (PDF) [file pgen.1006120.s017.pdf]

Table S3. Kolmogorov–Smirnov test was applied to the enrichments on the sequences with different similarities to the motif.

	-log10(p)<4	-log10(p): 4-5	-log10(p): 5-6	-log10(p)>6
-log10(p)<4				
-log10(p): 4-5	< 2.2e-16			
-log10(p): 5-6	< 2.2e-16	< 2.2e-16		
-log10(p) >6	< 2.2e-16	< 2.2e-16	8.4e-4	

Values show the Kolmogorov–Smirnov test p-values. Similarities to the motif were calculated with the FIMO tool, and categories are –log10(p-value) is smaller than 4, between 4 and 5, between 5 and 6, and larger than 6.
