# Supplementary material for: Expansion of GA Dinucleotide Repeats Increases the Density of CLAMP Binding Sites on the X-Chromosome to Promote Drosophila Dosage Compensation
Source: PLoS Genet. 2016 Jul 14;12(7):e1006120. doi: 10.1371/journal.pgen.1006120 (PMC4945028; doi:10.1371/journal.pgen.1006120)
Supplement: S4 Table — The Euclidean distance between all classes of motifs is shown. The Euclidean distance between the 8-bp cores and the flanking sequences are shown separately. (PDF) [file pgen.1006120.s018.pdf]

Table S4. Euclidean distance between motifs

Comparison	8-bp core	Flanking
PBM+ChIP+MRE+ vs PBM+ChIP+MRE-	0.080	0.212
PBM+ChIP+MRE+ vs PBM-ChIP-MRE+	0.091	0.117
PBM+ChIP+MRE+ vs PBM-ChIP-MRE-	0.268	0.264
PBM+ChIP+MRE- vs PBM-ChIP-MRE+	0.119	0.196
PBM+ChIP+MRE- vs PBM-ChIP-MRE-	0.235	0.200
PBM-ChIP-MRE+ vs PBM-ChIP-MRE-	0.247	0.210

The Euclidean distance between all classes of motifs is shown. The Euclidean distance between the 8-bp cores and the flanking sequences are shown separately.
