# Supplementary material for: Expansion of GA Dinucleotide Repeats Increases the Density of CLAMP Binding Sites on the X-Chromosome to Promote Drosophila Dosage Compensation
Source: PLoS Genet. 2016 Jul 14;12(7):e1006120. doi: 10.1371/journal.pgen.1006120 (PMC4945028; doi:10.1371/journal.pgen.1006120)
Supplement: S5 Table — Kolmogorov–Smirnov test was applied to the PBM intensities of probes with 8-bp core part and matched endogenous flank, with 8-bp core part and unmatched endogenous flank, with 8-bp core part and unmatched synthetic flank, without 8-bp core part, and with 8-bp core part and matched endogenous flank for 4 zinc finger protein. Values show the p-values. (PDF) [file pgen.1006120.s019.pdf]

Table S5. Kolmogorov-Smirnov test to compare binding to different flanking sequence categories.

Kolmogorov–Smirnov test (p-values)	8bp + matched endogenous flank	8bp + unmatched endogenous flank	8bp + unmatched synthetic flank	without 8bp core part	8bp + matched endogenous flank, 4ZF
8bp + matched endogenous flank					
8bp + unmatched endogenous flank	< 2.2e-16				
8bp + unmatched synthetic flank	< 2.2e-16	< 2.2e-16			
without 8bp core part	< 2.2e-16	< 2.2e-16	7.14e-11		
8bp + matched endogenous flank, 4ZF	< 2.2e-16	< 2.2e-16	< 2.2e-16	< 2.2e-16	

Kolmogorov–Smirnov test was applied to the PBM intensities of probes with 8-bp core part and matched endogenous flank, with 8-bp core part and unmatched endogenous flank, with 8-bp core part and unmatched synthetic flank, without 8-bp core part, and with 8-bp core part and matched endogenous flank for 4 zinc finger protein. Values show the p-values.
