# Supplementary material for: Expansion of GA Dinucleotide Repeats Increases the Density of CLAMP Binding Sites on the X-Chromosome to Promote Drosophila Dosage Compensation
Source: PLoS Genet. 2016 Jul 14;12(7):e1006120. doi: 10.1371/journal.pgen.1006120 (PMC4945028; doi:10.1371/journal.pgen.1006120)
Supplement: S6 Table — (PDF) [file pgen.1006120.s020.pdf]

Table S6. The count of probes that contain each number of GA-repeats.

GA repeats	# probes
1	65
2	1404
3	516
4	365
5	161
6	107
7	79
8	46
9	25
10	10
11+	17
