# Supplementary material for: Expansion of GA Dinucleotide Repeats Increases the Density of CLAMP Binding Sites on the X-Chromosome to Promote Drosophila Dosage Compensation
Source: PLoS Genet. 2016 Jul 14;12(7):e1006120. doi: 10.1371/journal.pgen.1006120 (PMC4945028; doi:10.1371/journal.pgen.1006120)
Supplement: S7 Table — Repeats are not overlapping, i.e. the repeats are assigned by the longest length. For the two replicates used in Fig 3D, these numbers are doubled. (PDF) [file pgen.1006120.s021.pdf]

Table S7. Number of GA-repeats in *D. melanogaster* autosomes (A) and the X-chromosome (X).

	A	X
GA*2	572439	119767
GA*3	34825	7760
GA*4	3703	1101
GA*5	877	330
GA*6	465	188
GA*7	309	146
GA*8	267	99
GA*9	147	82
GA*10	91	58
GA*11	77	28
GA*12+	106	68

Repeats are not overlapping, i.e. the repeats are assigned by the longest length. For the two replicates used in Fig 3D, these numbers are doubled.
