# Supplementary material for: Expansion of GA Dinucleotide Repeats Increases the Density of CLAMP Binding Sites on the X-Chromosome to Promote Drosophila Dosage Compensation
Source: PLoS Genet. 2016 Jul 14;12(7):e1006120. doi: 10.1371/journal.pgen.1006120 (PMC4945028; doi:10.1371/journal.pgen.1006120)
Supplement: S8 Table — Repeats with different lengths may be overlapping. (PDF) [file pgen.1006120.s022.pdf]

Table S8. Number of GA repeats in *D. melanogaster* chromosomes and individual chromosomal arms. Repeats with different lengths may be overlapping.

	X	A	2L	2R	3L	3R	4
**GA*2**	139219	649229	137531	152227	166567	186201	6703
**GA*3**	11273	44768	9384	10376	11774	12748	486
**GA*4**	2552	7065	1511	1610	1849	2063	32
**GA*5**	1191	2696	627	583	684	797	5
**GA*6**	752	1590	372	366	373	478	1
**GA*7**	517	1054	249	239	254	312	0
**GA*8**	352	710	186	152	167	205	0
**GA*9**	248	432	101	98	102	131	0
**GA*10**	160	278	69	59	66	84	0
**GA*11**	100	186	46	41	39	60	0
**GA*12**	72	109	21	26	25	37	0
**GA*13**	43	71	8	17	20	26	0
**GA*14**	32	53	6	12	13	22	0
**GA*15**	20	32	2	6	8	16	0
**GA*16**	13	19	2	4	4	9	0
**GA*17**	11	11	0	3	3	5	0
**GA*18**	10	9	0	3	2	4	0
**GA*19**	7	5	0	1	1	3	0
**GA*20**	6	3	0	1	1	1	0
**GA*21**	4	2	0	0	1	1	0
**GA*22**	4	2	0	0	1	1	0
**GA*23**	4	2	0	0	1	1	0
**GA*24**	4	2	0	0	1	1	0
**GA*25**	4	2	0	0	1	1	0
**GA*26**	3	2	0	0	1	1	0
**GA*27**	2	2	0	0	1	1	0
**GA*28**	2	1	0	0	0	1	0
