# Supplementary material for: Expansion of GA Dinucleotide Repeats Increases the Density of CLAMP Binding Sites on the X-Chromosome to Promote Drosophila Dosage Compensation
Source: PLoS Genet. 2016 Jul 14;12(7):e1006120. doi: 10.1371/journal.pgen.1006120 (PMC4945028; doi:10.1371/journal.pgen.1006120)
Supplement: S9 Table — (PDF) [file pgen.1006120.s023.pdf]

Table S9. Kolmogorov–Smirnov test was applied to the average distance values between S2 CLAMP ChIP-seq peaks.

		1 neighboring site			2 neighboring sites			3 neighboring sites			4 neighboring sites		
		CES	X	A	CES	X	A	CES	X	A	CES	X	A
1st neighbor	CES												
	X	1.64 e-03											
	A	4.54 e-03	< 2.2 e-16										
up to 2nd neighbor	CES	1.62 e-09	< 2.2 e-16	< 2.2 e-16									
	X	1.12 e-11	< 2.2 e-16	< 2.2 e-16	5.72 e-06								
	A	< 2.2 e-16	< 2.2 e-16	< 2.2 e-16	< 2.2 e-16	< 2.2 e-16							
up to 3rd neighbor	CES	< 2.2 e-16	< 2.2 e-16	< 2.2 e-16	4.86 e-11	< 2.2 e-16	< 2.2 e-16						
	X	< 2.2 e-16	< 2.2 e-16	< 2.2 e-16	< 2.2 e-16	< 2.2 e-16	< 2.2 e-16	2.86 e-11					
	A	< 2.2 e-16	< 2.2 e-16	< 2.2 e-16	< 2.2 e-16	< 2.2 e-16	< 2.2 e-16	< 2.2 e-16	< 2.2 e-16				
up to 4th neighbor	CES	< 2.2 e-16	< 2.2 e-16	< 2.2 e-16	< 2.2 e-16	< 2.2 e-16	< 2.2 e-16	5.67 e-14	1.37 e-11	< 2.2 e-16			
	X	< 2.2 e-16	< 2.2 e-16	< 2.2 e-16	< 2.2 e-16	< 2.2 e-16	< 2.2 e-16	< 2.2 e-16	< 2.2 e-16	5.86 e-11	8.30 e-13		
	A	< 2.2 e-16	< 2.2 e-16	< 2.2 e-16	< 2.2 e-16	< 2.2 e-16	< 2.2 e-16	< 2.2 e-16	< 2.2 e-16	< 2.2 e-16	< 2.2 e-16	< 2.2 e-16	
