# Supplementary material for: Expansion of GA Dinucleotide Repeats Increases the Density of CLAMP Binding Sites on the X-Chromosome to Promote Drosophila Dosage Compensation
Source: PLoS Genet. 2016 Jul 14;12(7):e1006120. doi: 10.1371/journal.pgen.1006120 (PMC4945028; doi:10.1371/journal.pgen.1006120)
Supplement: S10 Table — (PDF) [file pgen.1006120.s024.pdf]

Table S10. Kolmogorov–Smirnov test was applied to the average distance values between Kc CLAMP ChIP-seq peaks.

		1st neighbor			up to 2nd neighbor			up to 3rd neighbor			up to 4th neighbor		
		CES	X	A	CES	X	A	CES	X	A	CES	X	A
1st neighbor	CES												
	X	1.18 e-01											
	A	9.89 e-06	< 2.2 e-16										
up to 2nd neighbor	CES	1.44 e-15	< 2.2 e-16	< 2.2 e-16									
	X	< 2.2 e-16	< 2.2 e-16	< 2.2 e-16	4.26 e-05								
	A	< 2.2 e-16	< 2.2 e-16	< 2.2 e-16	< 2.2 e-16	< 2.2 e-16							
up to 3rd neighbor	CES	< 2.2 e-16	< 2.2 e-16	< 2.2 e-16	< 2.2 e-16	< 2.2 e-16	< 2.2 e-16						
	X	< 2.2 e-16	< 2.2 e-16	< 2.2 e-16	1.08 e-11	< 2.2 e-16	2.75 e-12	1.04 e-11					
	A	< 2.2 e-16	< 2.2 e-16	< 2.2 e-16	< 2.2 e-16	< 2.2 e-16	< 2.2 e-16	< 2.2 e-16	< 2.2 e-16				
up to 4th neighbor	CES	< 2.2 e-16	< 2.2 e-16	< 2.2 e-16	< 2.2 e-16	< 2.2 e-16	< 2.2 e-16	2.33 e-11	5.04 e-12	< 2.2 e-16			
	X	< 2.2 e-16	< 2.2 e-16	< 2.2 e-16	< 2.2 e-16	< 2.2 e-16	< 2.2 e-16	< 2.2 e-16	< 2.2 e-16	< 2.2 e-16	< 2.2 e-16		
	A	< 2.2 e-16	< 2.2 e-16	< 2.2 e-16	< 2.2 e-16	< 2.2 e-16	< 2.2 e-16	< 2.2 e-16	< 2.2 e-16	< 2.2 e-16	< 2.2 e-16	< 2.2 e-16	
