# Supplementary material for: Expansion of GA Dinucleotide Repeats Increases the Density of CLAMP Binding Sites on the X-Chromosome to Promote Drosophila Dosage Compensation
Source: PLoS Genet. 2016 Jul 14;12(7):e1006120. doi: 10.1371/journal.pgen.1006120 (PMC4945028; doi:10.1371/journal.pgen.1006120)
Supplement: S11 Table — (PDF) [file pgen.1006120.s025.pdf]

Table S11. Numbers of S2 and KC ChIP-seq peaks used in the average distance calculation are given.

	1st neighbor	up to 2nd neighbor	up to 3rd neighbor	up to 4th neighbor
S2 CES	337	606	864	1055
S2 X	2704	2704	2704	2704
S2 A	11607	11607	11607	11607
Kc CES	248	516	788	1037
Kc X	3220	3220	3220	3220
Kc A	11878	11878	11878	11878
