# Supplementary material for: Expansion of GA Dinucleotide Repeats Increases the Density of CLAMP Binding Sites on the X-Chromosome to Promote Drosophila Dosage Compensation
Source: PLoS Genet. 2016 Jul 14;12(7):e1006120. doi: 10.1371/journal.pgen.1006120 (PMC4945028; doi:10.1371/journal.pgen.1006120)
Supplement: S12 Table — (PDF) [file pgen.1006120.s026.pdf]

Table S12. Kolmogorov–Smirnov test was applied to the normalized distance values between S2 CLAMP ChIP-seq peaks.

		1st neighbor			up to 2nd neighbor			up to 3rd neighbor			up to 4th neighbor		
		CES	X	A	CES	X	A	CES	X	A	CES	X	A
1st neighbor	CES												
	X	2.88 e-03											
	A	1.16 e-02	1.43 e-05										
up to 2nd neighbor	CES	1.84 e-06	4.46 e-14	< 2.2 e-16									
	X	3.73 e-04	< 2.2 e-16	< 2.2 e-16	3.17 e-06								
	A	8.24 e-04	< 2.2 e-16	< 2.2 e-16	1.11 e-15	9.42 e-08							
up to 3rd neighbor	CES	1.51 e-08	< 2.2 e-16	< 2.2 e-16	3.70 e-02	2.83 e-11	< 2.2 e-16						
	X	6.55 e-09	< 2.2 e-16	< 2.2 e-16	5.61 e-07	4.81 e-07	1.05 e-12	9.82 e-12					
	A	4.32 e-09	< 2.2 e-16	< 2.2 e-16	< 2.2 e-16	< 2.2 e-16	< 2.2 e-16	< 2.2 e-16	1.98 e-12				
up to 4th neighbor	CES	9.38 e-12	< 2.2 e-16	< 2.2 e-16	3.46 e-05	5.55 e-16	< 2.2 e-16	8.17 e-02	1.48 e-13	< 2.2 e-16			
	X	1.50 e-13	< 2.2 e-16	< 2.2 e-16	7.30 e-07	< 2.2 e-16	< 2.2 e-16	2.74 e-12	9.35 e-04	9.99 e-16	6.10 e-13		
	A	7.49 e-13	< 2.2 e-16	< 2.2 e-16	< 2.2 e-16	< 2.2 e-16	< 2.2 e-16	< 2.2 e-16	< 2.2 e-16	2.07 e-09	< 2.2 e-16	< 2.2 e-16	
