# Supplementary material for: Expansion of GA Dinucleotide Repeats Increases the Density of CLAMP Binding Sites on the X-Chromosome to Promote Drosophila Dosage Compensation
Source: PLoS Genet. 2016 Jul 14;12(7):e1006120. doi: 10.1371/journal.pgen.1006120 (PMC4945028; doi:10.1371/journal.pgen.1006120)
Supplement: S13 Table — (PDF) [file pgen.1006120.s027.pdf]

Table S13. Kolmogorov–Smirnov test was applied to the normalized distance values between Kc CLAMP ChIP-seq peaks.

		1st neighbor			up to 2nd neighbor			up to 3rd neighbor			up to 4th neighbor		
		CES	X	A	CES	X	A	CES	X	A	CES	X	A
1st neighbor	CES												
	X	1.82 e-01											
	A	1.27 e-01	1.21 e-04										
up to 2nd neighbor	CES	1.36 e-07	4.55 e-15	< 2.2 e-16									
	X	3.01 e-07	< 2.2 e-16	< 2.2 e-16	1.32 e-04								
	A	1.63 e-05	< 2.2 e-16	< 2.2 e-16	1.32 e-07	4.71 e-03							
up to 3rd neighbor	CES	1.17 e-12	< 2.2 e-16	< 2.2 e-16	6.09 e-02	6.93 e-13	< 2.2 e-16						
	X	1.23 e-12	< 2.2 e-16	< 2.2 e-16	2.59 e-04	5.45 e-09	< 2.2 e-16	1.05 e-11					
	A	1.74 e-10	< 2.2 e-16	< 2.2 e-16	9.64 e-08	1.31 e-13	< 2.2 e-16	< 2.2 e-16	1.16 e-05				
up to 4th neighbor	CES	2.83 e-14	< 2.2 e-16	< 2.2 e-16	9.97 e-03	< 2.2 e-16	< 2.2 e-16	4.34 e-01	< 2.2 e-16	< 2.2 e-16			
	X	4.44 e-16	< 2.2 e-16	< 2.2 e-16	8.03 e-05	< 2.2 e-16	< 2.2 e-16	6.49 e-11	1.07 e-04	3.70 e-11	3.33 e-16		
	A	5.37 e-14	< 2.2 e-16	< 2.2 e-16	3.38 e-09	< 2.2 e-16	< 2.2 e-16	< 2.2 e-16	2.43 e-11	1.32 e-08	< 2.2 e-16	9.11 e-09	
