# Supplementary material for: Expansion of GA Dinucleotide Repeats Increases the Density of CLAMP Binding Sites on the X-Chromosome to Promote Drosophila Dosage Compensation
Source: PLoS Genet. 2016 Jul 14;12(7):e1006120. doi: 10.1371/journal.pgen.1006120 (PMC4945028; doi:10.1371/journal.pgen.1006120)
Supplement: S14 Table — Conservation of CLAMP relative to D. melanogaster was determined for both the full-length protein (FL) and the DNA binding domain (DBD) alone. (PDF) [file pgen.1006120.s028.pdf]

Table S14. Conservation of CLAMP across species .

Species	FL % Identity	FL % Similarity	DBD % Identity	DBD % Similarity
D. melanogaster	100.0	100.0	100.0	100.0
D. miranda	92.6	95.9	99.4	100.0
A. gambiae	50.5	59.0	89.0	93.9

Conservation of CLAMP relative to *D. melanogaster* was determined for both the full-length protein (FL) and the DNA binding domain (DBD) alone.
