# Supplementary material for: Expansion of GA Dinucleotide Repeats Increases the Density of CLAMP Binding Sites on the X-Chromosome to Promote Drosophila Dosage Compensation
Source: PLoS Genet. 2016 Jul 14;12(7):e1006120. doi: 10.1371/journal.pgen.1006120 (PMC4945028; doi:10.1371/journal.pgen.1006120)
Supplement: S15 Table — (PDF) [file pgen.1006120.s029.pdf]

Table S15. Number of matches to the CLAMP PBM motif per Mb on individual chromosomal arms.

D. melanogaster		***D. miranda***		***A. gambiae***	
chr2L	91.6	chr2	387.5	chr2L	409.7
chr2R	88.2	chr4	507.3	chr2R	416.5
chr3L	90.6	chr5	136.0	chr3L	334.1
chr3R	91.4	neoX	478.0	chr3R	372.3
chr4	11.9	chrXL	645.4	chrX	870.0
chrX	202.8	chrXR	729.0		
