# Supplementary material for: Expansion of GA Dinucleotide Repeats Increases the Density of CLAMP Binding Sites on the X-Chromosome to Promote Drosophila Dosage Compensation
Source: PLoS Genet. 2016 Jul 14;12(7):e1006120. doi: 10.1371/journal.pgen.1006120 (PMC4945028; doi:10.1371/journal.pgen.1006120)
Supplement: S16 Table — Repeats may be overlapping. (PDF) [file pgen.1006120.s030.pdf]

Table S16. Number of GA repeats in *D. miranda* chromosomes. Repeats may be overlapping.

	**XL**	**XR**	**3**	**A (2,4,5)**	**2**	**4**	**5**
**GA*2**	92978	130682	83961	252853	130383	117364	5106
**GA*3**	13816	20057	10967	34014	17372	16151	491
**GA*4**	4819	7111	3574	10618	5407	5058	153
**GA*5**	2475	3674	1753	5126	2663	2439	24
**GA*6**	1637	2416	1147	3207	1645	1542	20
**GA*7**	1104	1657	809	2083	1049	1023	11
**GA*8**	749	1138	564	1378	681	689	8
**GA*9**	519	792	405	917	443	467	7
**GA*10**	379	562	272	648	301	342	5
**GA*11**	265	415	183	458	210	243	5
**GA*12**	193	286	130	344	151	188	5
**GA*13**	137	218	92	244	102	138	4
**GA*14**	94	160	64	178	68	107	3
**GA*15**	66	111	46	129	50	76	3
**GA*16**	51	81	37	98	38	57	3
**GA*17**	40	67	28	76	28	46	2
**GA*18**	29	48	22	56	22	32	2
**GA*19**	23	40	13	35	11	23	1
**GA*20**	18	32	9	29	8	21	0
**GA*21**	16	26	8	24	7	17	0
**GA*22**	15	24	5	21	7	14	0
**GA*23**	12	18	3	17	7	10	0
**GA*24**	10	17	3	15	6	9	0
**GA*25**	9	15	2	13	5	8	0
**GA*26**	8	12	2	11	4	7	0
**GA*27**	6	12	2	7	1	6	0
**GA*28**	5	9	1	7	1	6	0
