# Supplementary material for: Expansion of GA Dinucleotide Repeats Increases the Density of CLAMP Binding Sites on the X-Chromosome to Promote Drosophila Dosage Compensation
Source: PLoS Genet. 2016 Jul 14;12(7):e1006120. doi: 10.1371/journal.pgen.1006120 (PMC4945028; doi:10.1371/journal.pgen.1006120)
Supplement: S17 Table — Repeats may be overlapping. (PDF) [file pgen.1006120.s031.pdf]

Table S17 Number of GA repeats in *A. gambiae* chromosomes. Repeats may be overlapping.

	X	A	2L	2R	3L	3R
**GA*2**	87061	655817	159887	192336	132715	170879
**GA*3**	14166	79426	19639	23604	15466	20717
**GA*4**	6356	26736	6686	8331	4955	6764
**GA*5**	4097	15820	3991	4979	2843	4007
**GA*6**	3034	10851	2749	3488	1928	2686
**GA*7**	2273	7687	1954	2514	1341	1878
**GA*8**	1732	5522	1397	1835	982	1308
**GA*9**	1343	4048	1024	1360	707	957
**GA*10**	1063	3110	790	1036	539	745
**GA*11**	867	2498	637	841	420	600
**GA*12**	731	2102	534	705	357	506
**GA*13**	616	1808	451	612	304	441
**GA*14**	522	1554	381	526	272	375
**GA*15**	458	1355	329	456	247	323
**GA*16**	404	1182	286	400	217	279
**GA*17**	352	1055	264	355	194	242
**GA*18**	315	921	230	310	168	213
**GA*19**	270	804	207	267	141	189
**GA*20**	233	687	179	228	113	167
**GA*21**	196	594	160	192	102	140
**GA*22**	168	514	133	171	93	117
**GA*23**	146	450	116	149	82	103
**GA*24**	131	399	104	127	75	93
**GA*25**	117	344	88	110	65	81
**GA*26**	96	304	76	96	55	77
**GA*27**	89	258	62	78	52	66
**GA*28**	74	228	53	68	48	59
